# GDC: Integration of Multi‐Omic and Phenotypic Resources to Unravel the Genetic Pathogenesis of Hearing Loss

**DOI:** 10.1002/advs.202408891

**Published:** 2025-03-16

**Authors:** Hui Cheng, Xuegang Wang, Mingjun Zhong, Jia Geng, Wenjian Li, Kanglu Pei, Jing Wang, Lanchen Wang, Yu Lu, Jing Cheng, Fengxiao Bu, Huijun Yuan

**Affiliations:** ^1^ Department of Oto‐Rhino‐Laryngology West China Hospital of Sichuan University Chengdu 610000 China; ^2^ Institute of Rare Diseases West China Hospital of Sichuan University Chengdu 610044 China

**Keywords:** database, gene expression, hearing loss, mouse phenotype, machine learning

## Abstract

Effective research and clinical application in audiology and hearing loss (HL) require the integration of diverse data, yet the absence of a dedicated database impedes understanding and insight extraction in HL. To address this, the Genetic Deafness Commons (GDC) is developed by consolidating extensive genetic and genomic data from 51 public databases and the Chinese Deafness Genetics Consortium. This repository comprises 5 983 613 variants across 201 HL genes, revealing the genetic landscape of HL and identifying six novel mutational hotspots within the DNA‐binding domains of transcription factors. Comparative phenotypic analyses highlighted considerable disparities between human and mouse models. Among the 201 human HL genes, 133 exhibit hearing abnormalities in mice; 35 have been tested in mice without exhibiting a hearing loss phenotype; and 33 lack auditory testing data. Moreover, gene expression analyses in the cochleae of mice, humans, and rhesus macaques demonstrated a notable correlation (R^2^ 0.718–0.752). Utilizing gene expression, function, pathway, and phenotype data, a SMOTE‐Random Forest model identified 18 candidate HL genes, including *TBX2*, newly confirmed as an HL gene. As a comprehensive and unified repository, the GDC advances audiology research and practice by improving data accessibility and usability, ultimately fostering deeper insights into hearing disorders.

## Graphical abstract

Overview of the Genetic Deafness Commons (GDC), integrating data from the Chinese Deafness Genetics Consortium (CDGC) and 51 public databases. The GDC provides tools for variant search, functional predictions, and gene‐disease visualization, offering insights into 201 hearing loss genes and facilitating novel gene discovery and clinical applications.

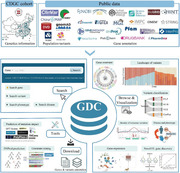

## 1 Introduction

Hearing loss (HL) is the most common sensory deficit and one of the most common congenital abnormalities. Estimates indicate that among every 1000 newborns screened, 1.1–3.5 will have HL.^[^ ^1^, ^2^ ^]^ The etiology of HL is multifactorial, encompassing genetic defects, physical trauma, infections, drug toxicity, noise exposure, and aging, among other factors.^[^ ^3^, ^4^, ^5^ ^]^ Genetic predispositions play a pivotal role in congenital and early‐onset HL, characterized by significant genetic and phenotypic heterogeneity, with more than 200 genes identified thus far.^[^ ^6^, ^7^, ^8^ ^]^

Current research and clinical applications in audiology and hearing disorders, such as novel HL gene identification, auditory mechanism exploration, research on the auditory system development, variant interpretation, and genetic diagnosis, and the development of gene therapies, hold the promise of translating individual genomic data into clinically relevant information to aid in disease diagnostics and facilitate personalized therapeutic decision making.^[^ ^9^, ^10^, ^11^, ^12^, ^13^, ^14^, ^15^ ^]^ These efforts depend critically on the integration of vast data and knowledge accumulated across numerous databases and repositories. This integration includes gene/region‐level annotations detailing genomic features (e.g., UCSC genome browsers^[^ ^16^ ^]^ and ENSEMBL^[^ ^17^ ^]^), transcriptional information (e.g., gEAR^[^ ^18^ ^]^ and Genotype‐Tissue Expression (GTEx)^[^ ^19^ ^]^), gene/protein functions (e.g., UniProtKB^[^ ^20^ ^]^ and Gene Ontology (GO)^[^ ^21^ ^]^) and structures (e.g., InterPro^[^ ^22^ ^]^ and PDB^[^ ^23^ ^]^), pathway information (e.g., Reactome^[^ ^24^ ^]^ and Kyoto Encyclopedia of Genes and Genomes (KEGG)^[^ ^25^ ^]^), gene‐gene interactions (e.g., STRING^[^ ^26^ ^]^ and BioGrid^[^ ^27^ ^]^), and gene‐drug interactions (e.g., PharmGKB^[^ ^28^ ^]^ and DGIdb^[^ ^29^ ^]^). Variant annotations also play a pivotal role in interpreting the effects of molecular processes and disease causation, including population frequencies (e.g., gnomAD,^[^ ^30^ ^]^ ChinaMAP,^[^ ^31^ ^]^ and BBJ^[^ ^32^ ^]^), genotype‐phenotype correlations and pathogenicity classifications (e.g., ClinVar,^[^ ^33^ ^]^ HGMD,^[^ ^34^ ^]^ and Deafness Variation Database (DVD)^[^ ^6^ ^]^), in silico function predictions (e.g., CADD,^[^ ^35^ ^]^ REVEL,^[^ ^36^ ^]^ and SpliceAI^[^ ^37^ ^]^), and automated literature mining (e.g., LitVar^[^ ^38^ ^]^ and Variant2Literature^[^ ^39^ ^]^). Additionally, disease and phenotype information extracted from literature through expert curation includes standardized disease/phenotype descriptions (e.g., Human Phenotype Ontology (HPO)^[^ ^40^ ^]^ and MedlinePlus^[^ ^41^ ^]^), disease‐gene correlation (e.g., OMIM^[^ ^42^ ^]^ and ClinGen^[^ ^43^ ^]^), and animal models (e.g., Mouse Genome Informatics (MGI)^[^ ^44^ ^]^). Together, these diverse databases provide essential support for researchers to explore the complexities of genetic and molecular mechanisms underlying audiology and hearing disorders, offering crucial insights into broader biological processes, disease pathogenesis, and potential therapeutic interventions.

However, the lack of a dedicated, comprehensive database for hearing research has significantly hindered the compilation and integration of these resources. Existing hearing‐related databases like DVD and gEAR cater to niche aspects of hearing loss research, with DVD focusing on variant classification within HL genes and gEAR on gene expression in the cochlea. Information is often scattered across specialized catalogs tailored to specific fields, particular model organisms, or specific techniques, resulting in heterogeneous data vocabularies, ontologies, and formats. It complicates efforts to fully reconcile and integrate the data, posing significant challenges in understanding the landscape of hearing loss, identifying and prioritizing relevant information, and consequently impeding the extraction of meaningful insights.

To bridge the gap, we established Genetic Deafness Commons (GDC, http://gdcdb.net/), a standardized database and knowledge base that comprehensively consolidates and integrates genetic and genomic data from both public and in‐house sources. The GDC leveraged over 51 public databases to provide extensive information on HL genes, variants, and phenotypes. Additionally, it integrates genetic findings from 22 125 HL cases from the Chinese Deafness Genetics Consortium (CDGC), offering real‐world patient cohort data to support variant interpretation and curation. Utilizing the extensive dataset of GDC, this study conducts a thorough analysis of the genetic architecture of HL genes and variants, uncovering multiple new patterns to improve the efficacy of variant pathogenicity interpretation and genetic diagnosis. By integrating public gene expression data from mice and in‐house data from rhesus macaques, this study applied the machine learning method to identify a set of new candidate HL genes, demonstrating the significant value of GDC in deciphering the genetic and molecular foundations of audiology and hearing‐related conditions.

## 2 Results

### 2.1 Overview of GDC

**Figure 1:**
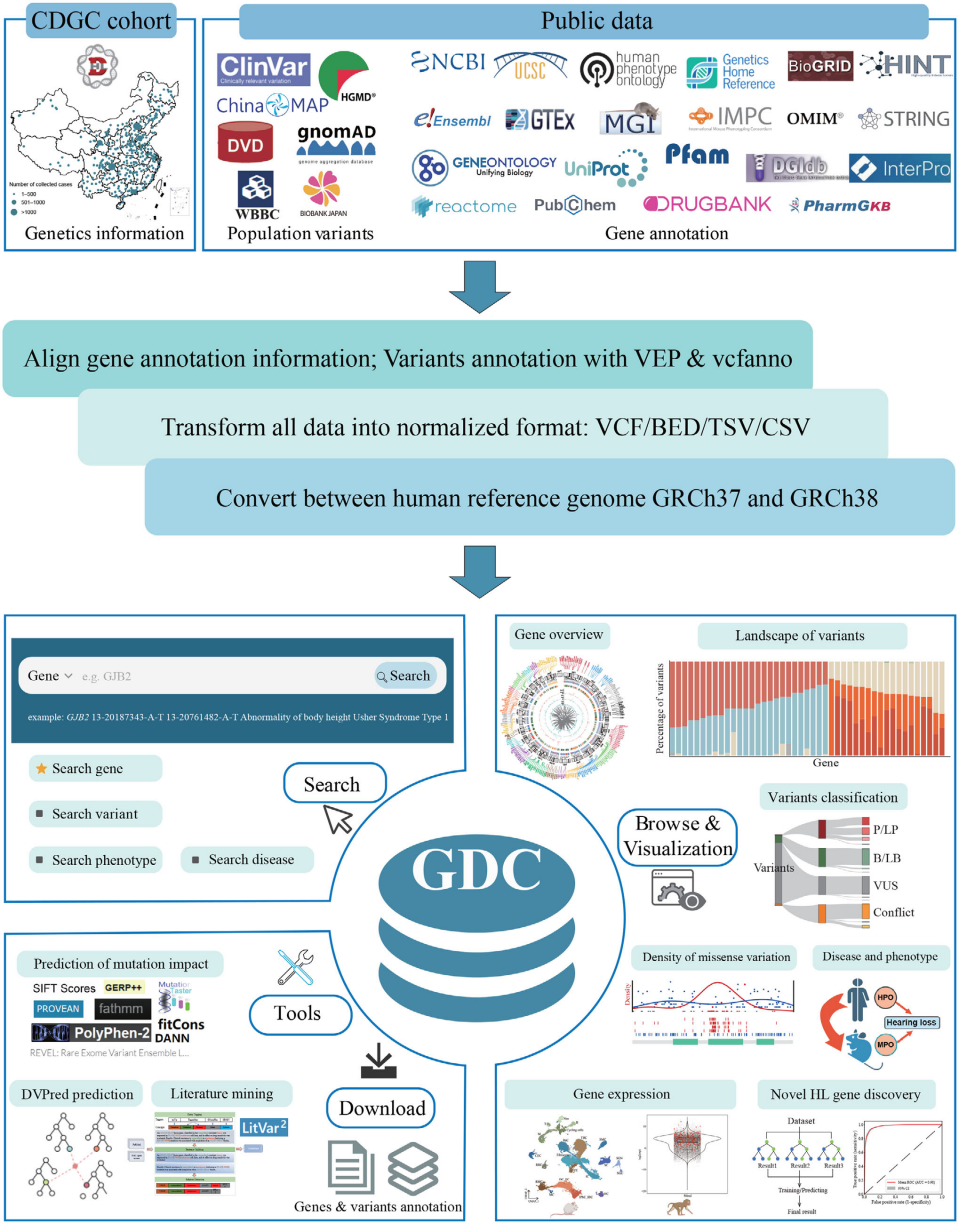
Construction of the Genetic Deafness Commons (GDC). The data from 51 public databases and the Chinese Deafness Genetics Consortium were collected. A series of data processing, including data alignment, gene and variant annotation, format transformation, and lifting over between human reference genomes GRCh37 and GRCh38, were applied to integrate and harmonize data in GDC. All data on GDC can be accessed online or downloaded. All the above logos are from official databases. Illustrations of humans, mice, and rhesus monkeys were created with Biorender.com.

The current version of the GDC amalgamated data from 51 public sources and the CDGC project, incorporating 5 983 613 variants across 201 reported HL genes (**Figure** **1** and Table S1, Supporting Information). GDC encompasses detailed disease information, gene expression patterns, annotations related to gene functions and pathways, protein structures and domains, gene‐gene and gene‐drug interactions, as well as other essential data. Additionally, GDC introduces multiple specialized annotations to enhance understanding of each variant, including allele frequencies from 14 populations of five large genome sequencing studies, variant pathogenicity classifications from four sources, 20 prediction scores for functional damage, genotype‐phenotype correlations, and variant‐based literature mining results. Comprehensive data sources are detailed in Table S2 (Supporting Information) to support further exploration. Furthermore, GDC offers a user‐friendly query interface to ensure that information is readily accessible in various formats such as graphical representations, tables, and downloadable files, facilitating easy search and navigation for researchers investigating any HL‐related variants and genes.

**Figure 2:**
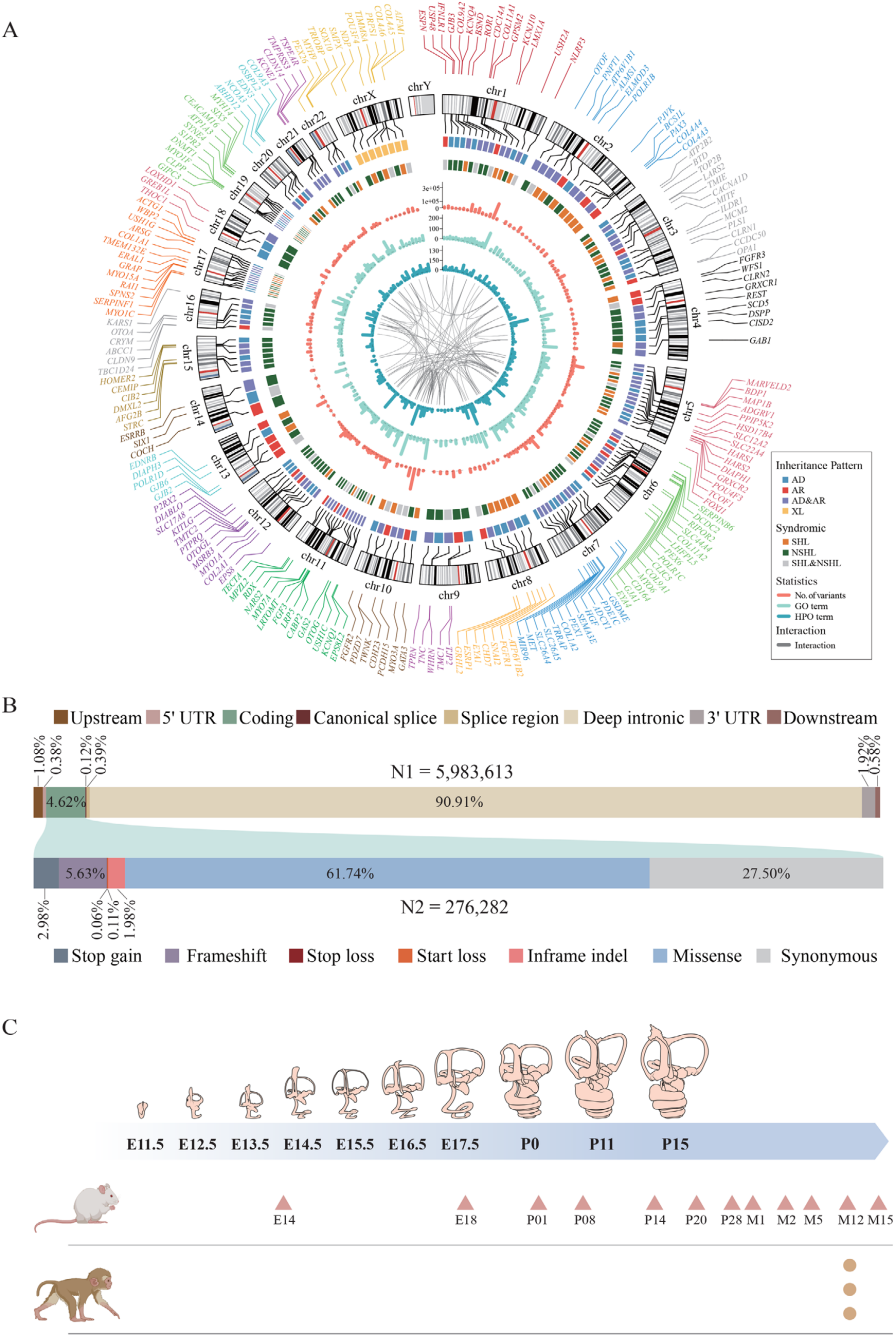
Summary of genetic and genomic information in GDC. A) Summarization of 201 HL genes. The outer circle is the genomic location of 201 genes. Genes located on different chromosomes are distinguished by color. Inner circles were inheritance patterns, syndromic HL or not, statistics of variants, GO term, HPO term, and gene‐gene interactions, respectively. B) The total number and proportion of variants observed classified by genomic location. Variants in coding regions were further classified by functional consequence. C) Developmental time points of gene expression data from cochleae of mice and rhesus macaques that were integrated by GDC.

Of the 201 HL genes in the GDC, 139 were associated with non‐syndromic HL (NSHL) and 93 with syndromic HL (SHL), linking to 92 different disorders including Usher syndrome, Alport syndrome, and Waardenburg syndrome (**Figure** **2**A). Notably, 31 genes were implicated in both NSHL and SHL. In terms of inheritance patterns, 95 genes were linked to autosomal dominant (AD), 128 to autosomal recessive (AR), eight to X‐linked (XL), and one to mitochondrial (MT) inheritance patterns. Furthermore, 31 genes exhibit both AD and AR inheritance patterns. These genes are annotated with 7656 functional terms (with a median of 27 terms per gene) and 8695 phenotypic terms (with a median of 19 terms per gene).

Within the exons, introns, and 1 kb upstream and downstream regions of the 201 HL genes, 5983613 genetic variations were collated in the GDC from multiple sources including CDGC (v202407, *n* = 217 915), gnomAD (v3.1, *n* = 4 973 216), ChinaMAP (v1, *n* = 997 781), DVD (v9, *n* = 2 100 721), ClinVar (20 230 702, *n* = 121 439), and HGMD (2023v2, *n* = 24 470). Of all variants, 4.63% were located in exonic and adjacent (±8 bp) intronic regions. Missense variants constitute the majority of such variants at 61.74%. The next most prevalent types are synonymous variants (27.5%) followed by indels (5.63% frameshift and 1.98% in‐frame), stop gain (2.98%), 5′ UTR (0.38%), canonical splice sites (±2 bp of an intron, 0.12%), splice regions (±3‐8 bp of an intron, 0.39%), 3′ UTR (1.92%), up/downstream regions (1.68%), and start/stop loss (<0.2%) (Figure 2B). Most variants were extremely rare, with the GDC dataset containing 1 436 357 (24%) variants not reported in gnomAD. Additionally, 2 338 525 (39.06%) variants in the GDC dataset are singletons in gnomAD, with doubletons, tripletons, and quadruplets accounting for 10.72%, 5.15%, and 3.08% respectively, and 639113 variants (10.67%) have a minor allele frequency (MAF) < 0.0002 and allele count (AC) > 4.

Furthermore, GDC incorporated extensive gene expression data from both human and model animals. This included bulk RNA‐seq data of 54 human tissues sourced from the GTEx database, single‐cell RNA‐seq data at 12 developmental stages of the mouse cochlea from five public repositories (GSE172110, GSE182202, GSE181454, GSE202920, and CRA004814)^[^ ^45^, ^46^, ^47^, ^48^, ^49^ ^]^ and in‐house bulk RNA‐seq data from three adult rhesus macaque cochlea (Figure 2C). These datasets enable the GDC to provide a detailed and dynamic view of gene expression across different species and developmental stages, significantly enhancing the potential for discoveries in audiology and hearing disorders.

### 2.2 Variant Pathogenicity Classification and Curation

**Figure 3:**
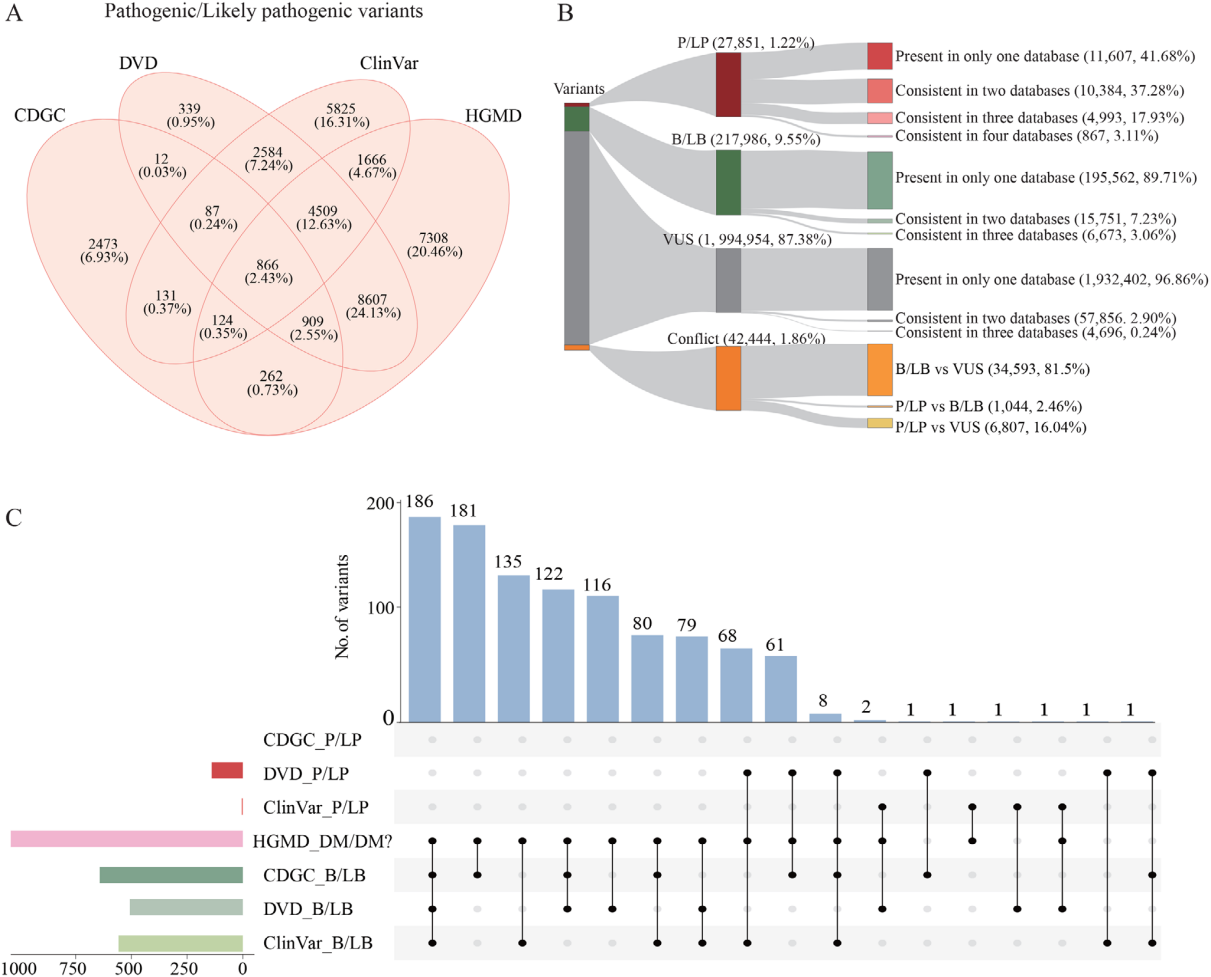
Variant pathogenicity classification from varied sources. A) Shared and unique P/LP variants collected from DVD, ClinVar, HGMD, and CDGC datasets. B) Comparative overview of variant classification in GDC and the consistency among the databases. C) P/LP and B/LB conflicts across DVD, ClinVar, HGMD, and CDGC. Bar plot on left side shows total number of conflicted variant classifications for each dataset.

Across the CDGC, DVD, ClinVar, and HGMD databases, 38.18% (*n* = 2 284 692) of all GDC variants were classified as pathogenic (P), likely pathogenic (LP), benign (B), likely benign (LB), or variants with uncertain significance (VUS). A total of 35 702 variants were reported as P/LP by at least one source (**Figure** **3**A). Among these variants, 16 244 (45.5%) were consistently classified as P/LP across two or more sources, whereas 11 607 (32.51%) P/LP variants were reported in only one source. Conversely, 7851 (21.99%) variants showed medically significant classification conflicts, shifting between P/LP and B/LB, or VUS, indicating the need for further validation using more extensive patient data (Figure 3B). Among the databases, HGMD exhibited the highest incidence of 1040 conflicts between P/LP and B/LB classifications, as shown in Figure 3C and Figure S1 (Supporting Information). Remarkably, 640 variants previously classified as “P/LP” (“DM” or “DM?”) in HGMD were reclassified to B/LB by CDGC. This reclassification accounted for 467 variants due to an MAF > 0.5% for AR or MAF > 0.1% for AD, 134 variants due to relatively high MAF combined with benign computational predictions, 35 AD‐associated variants detected in multiple CDGC controls, and 4 variants based on other combined evidence. This highlights the importance and efficacy of incorporating data from diverse and underrepresented populations in variant interpretation.

### 2.3 Analyzing Pathogenic Variants for Insights into Gene Function and Tolerance

**Figure 4:**
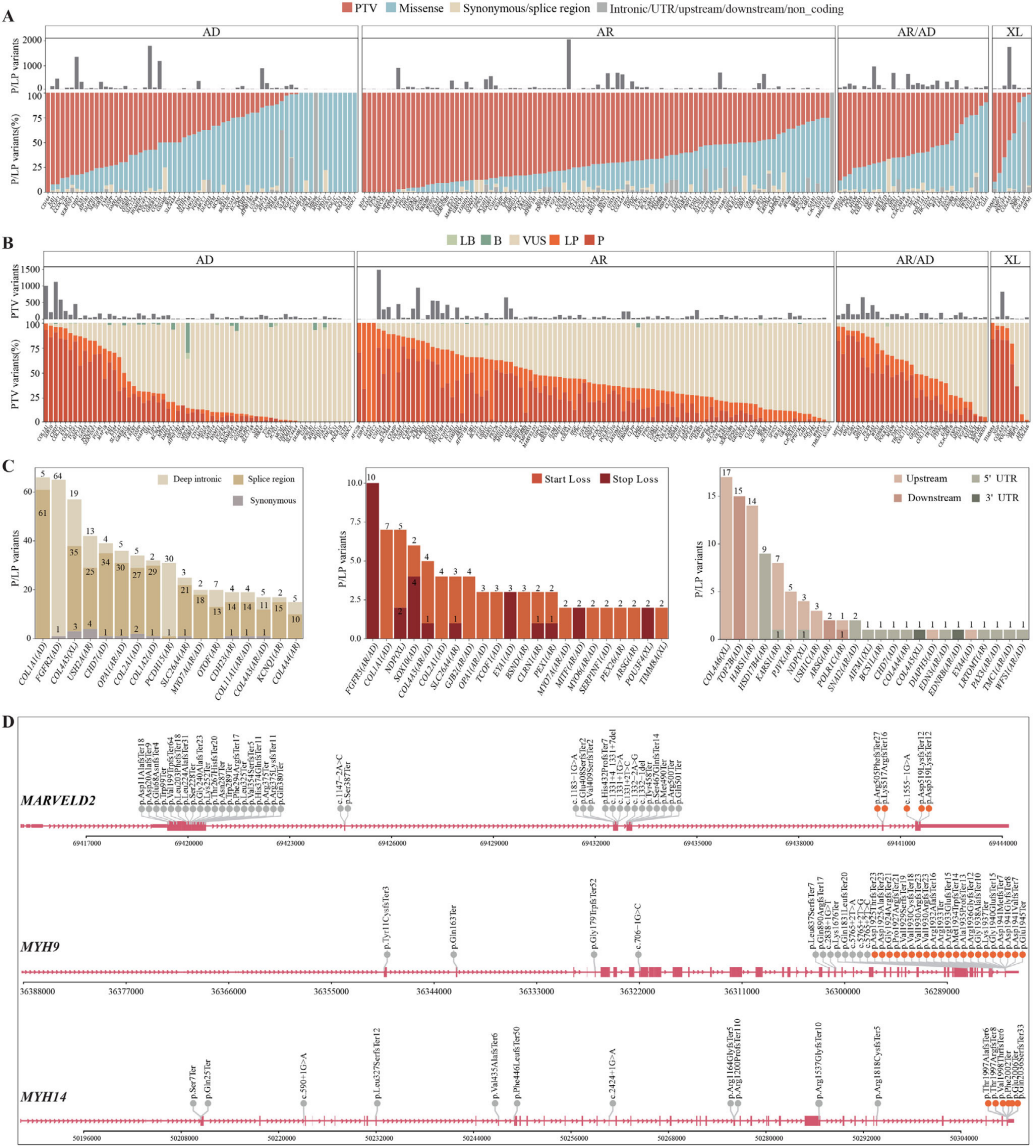
Genetic landscape of HL‐associated genes. A) Variant types of P/LP variants across 201 HL genes. The barplot above indicates the number of variants. Abbreviations: AD, Autosomal dominant; AR, Autosomal recessive; XL, X‐linked; PTV, Protein Truncating Variant. B) Pathogenicity classification of PTVs across 201 HL genes. C) Distribution of rare types of pathogenic variants, including deep intronic, synonymous, splice region, start loss, stop loss, UTRs, and intergenic. D) Distribution of PTVs in genes enriched with NMD‐escape PTVs. The orange circle indicates that the variants are within the last exon or the final 50 base pairs of the penultimate exon.

Protein truncating variants (PTVs), including stop‐gain, frameshift, start loss, stop loss, and canonical splice site changes, are expected to result in a complete loss of function (LoF) of the affected transcripts and are considered potentially deleterious.^[^ ^50^ ^]^ Within the protein‐coding genes included in the GDC, the count of PTVs ranged from 6 to 1886. A correlation between PTV count and coding region size was observed (R^2^ = 0.72, Figure S2 and Table S3, Supporting Information). However, genes such as *AIFM1*, *MAP1B*, and *USP48* exhibited a significantly lower PTV ratio, suggesting an intolerance to PTVs in these genes. A total of 31558 PTVs were classified by CDGC, DVD, ClinVar, or HGMD. Notably, the majority of the PTVs classified as VUS (*n* = 6785) were contributed by DVD (77.47%). Analysis of the variant types among P/LP classifications for each gene revealed that PTVs constitute more than half of P/LP variants in 127 genes (67.91%) (**Figure** **4**A). All P/LP variants in the gene *EPS8* (*n* = 13), *SYNE4* (*n* = 12), *MPZL2* (*n* = 11), *GRXCR2* (*n* = 5), *BDP1* (*n* = 3), *GAS2* (*n* = 2), *CLDN9* (*n* = 1), and *CD164* (*n* = 1) were PTVs, indicating that LoF is likely the disease mechanism in these genes. In 52 genes (27.96%), more than half of the P/LP variants were missense (Figure 4A). Specifically, in genes like *DIABLO*, *ELMOD3*, *IFNLR1*, *MYO1C*, *P2RX2*, *PDE1C*, *PLS1*, *POLR1B*, *S1PR2*, *SCD5*, *THOC1*, and *WBP2*, all P/LP variants were missense. By literature review, a gain of function (GoF) mechanism was only reported in *DIABLO*,^[^ ^51^ ^]^ suggesting further exploration of GoF as the probable disease mechanism across these genes. By analyzing the rates of P/LP variants among PTVs for each gene, the distribution of genes across different inheritance patterns is visualized (Figure 4B). Genes with an established LoF mechanism, such as *POU4F3*, *CHD7*, *MITF*, *EYA1*, and *EYA4*, were located in a quadrant where both the P/LP ratio of PTVs and the PTV ratio of P/LP variants exceed 50% (Figure S3, Supporting Information). Conversely, genes tolerant to LoF, such as *DIAPH3* and *COCH*, are found in a quadrant where both ratios are below 50%. It is noteworthy that genes with potentially lethal LoF variants, like *ACTG1* and *AIFM1*, also fall into this category.

In addition, rare types of pathogenic variants were observed in multiple genes (Figure 4C). For instance, pathogenic splicing variants are prevalent in genes such as *COL1A1*, *FGFR2*, *COL4A5*, and *USH2A*. Loss‐of‐start and loss‐of‐stop variants are typically found in genes *FGFR3*, *COL1A1*, and *NDP*. Furthermore, pathogenic variants affecting upstream regions, untranslated regions (UTRs), or downstream non‐coding regions are observed in genes *COL4A6*, *TOP2B*, and *HARS1*.

In certain genes, *MARVELD2*, *MYH9*, *MYH14*, *CABP2*, *CLIC5*, *HOMER2*, *SPATA5L1*, and *WFS1*, pathogenic PTVs were abundantly identified within the last exon or the final 50 base pairs of the penultimate exon (Figure 4D; Figure S4, Supporting Information), regions predicted to potentially escape nonsense‐mediated mRNA decay (NMD). The pathogenicity of 3′‐end PTVs in these genes requires careful evaluation, given their potentially significant impact on protein activity and stability.

### 2.4 Identification of “Hotspots” for Pathogenic Missense Variants

**Figure 5:**
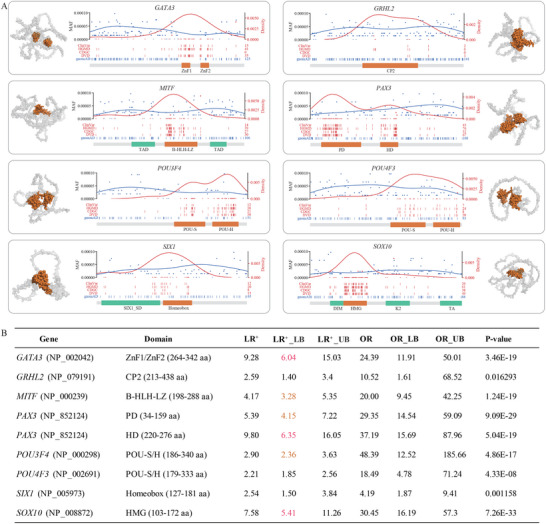
Identification of gene domains for enrichment of pathogenic missense variants. A) Distribution of P/LP missense variants across genes. Red curves are the density for P/LP variants reported in ClinVar, HGMD, DVD, and CDGC cohort. Blue curves are for variants identified in the gnomAD population. The horizontal bar represents protein domains, with the orange color indicating DNA‐binding domains. Abbreviations: ZnF, Zinc finger; CP2, Grh/CP2 DNA‐binding (DB) domain profile; TAD, Transactivation Domin; bHLH‐LZ, Basic helix‐loop‐helix, Leucine‐zipper; PD, “Paired box” domain; HD, Homeodomain; POU‐S, POU‐specific domain; POU‐H, POU‐homeodomain; SIX1_SD, Transcriptional regulator, SIX1, N‐terminal SD domain; DIM, Dimerization; HMG, High mobility group box; K2, Context‐dependent transactivation (SOX E group conserved) domain; TA, Transactivation. B) Statistical analysis of likelihood ratio (LR) and odds ratio (OR) for a missense variant being P/LP in these domains.

We further evaluate the distribution of missense P/LP variants using Gaussian kernel density estimation, overlapping with protein domains to identify hotspots of disease‐related missense mutations. By integrating various sources, we identified 8787 missense P/LP variants in 201 HL genes (ranging from 1 to 785 per gene). Significant enrichment of P/LP missense variants was predominantly found in transcription factor genes, including *GATA3* (NP_002042: p.264‐342), *GRHL2* (NP_079191: p.213‐438), *LMX1A* (NP_796372: p.196‐252), *MITF* (NP_000239: p.198‐288), *PAX3* (NP_852124: p.34‐159; p.220‐276), *POU3F4* (NP_000298: p.186‐340), *POU4F3* (NP_002691: p.179‐333), *REST* (NP_001350382: p.159‐412), *SIX1* (NP_005973: p.127‐181), and *SOX10* (NP_008872: p.103‐172) (Table S4, Supporting Information). Remarkably, these enriched regions were all DNA‐binding domains (**Figure** **5**A; Figure S5, Supporting Information). The exceptions in transcription factor genes were *FOXI1*, *SIX5*, and *SNAI2*, likely due to the limited number of identified P/LP variants. We calculated the positive likelihood ratio for a missense variant being P/LP in these hotspot regions. The lower boundary of the 95% confidence interval of the positive likelihood ratio (LR+_LB) ranged from 0.26 to 9.75 (Figure 5B). Compared to the thresholds for pathogenic evidence as suggested by Tavtigian et al.,^[^ ^52^ ^]^ hotspots in *PAX3, SOX10*, and *GATA3* met the moderate level (LR+_LB > 4.3) of the American College of Medical Genetics and Genomics (ACMG) and the Association for Molecular Pathology (AMP) pathogenic evidence. *LMX1A*, *POU3F4*, and *MITF* fulfilled the supporting level (LR+_LB > 2.08) for pathogenicity according to the same criteria. Additionally, missense variants of *KCNQ1* and *KCNQ4* were significantly enriched in the ion transport domain (LR+_LB = 2.08) and P‐loop domain (LR+_LB = 9.78), respectively, consistent with previous reports.^[^ ^53^ ^]^ No significant missense variant enrichment was observed in other genes.

### 2.5 Discrepancies of HL Phenotype Between Human and Mouse Models

**Figure 6:**
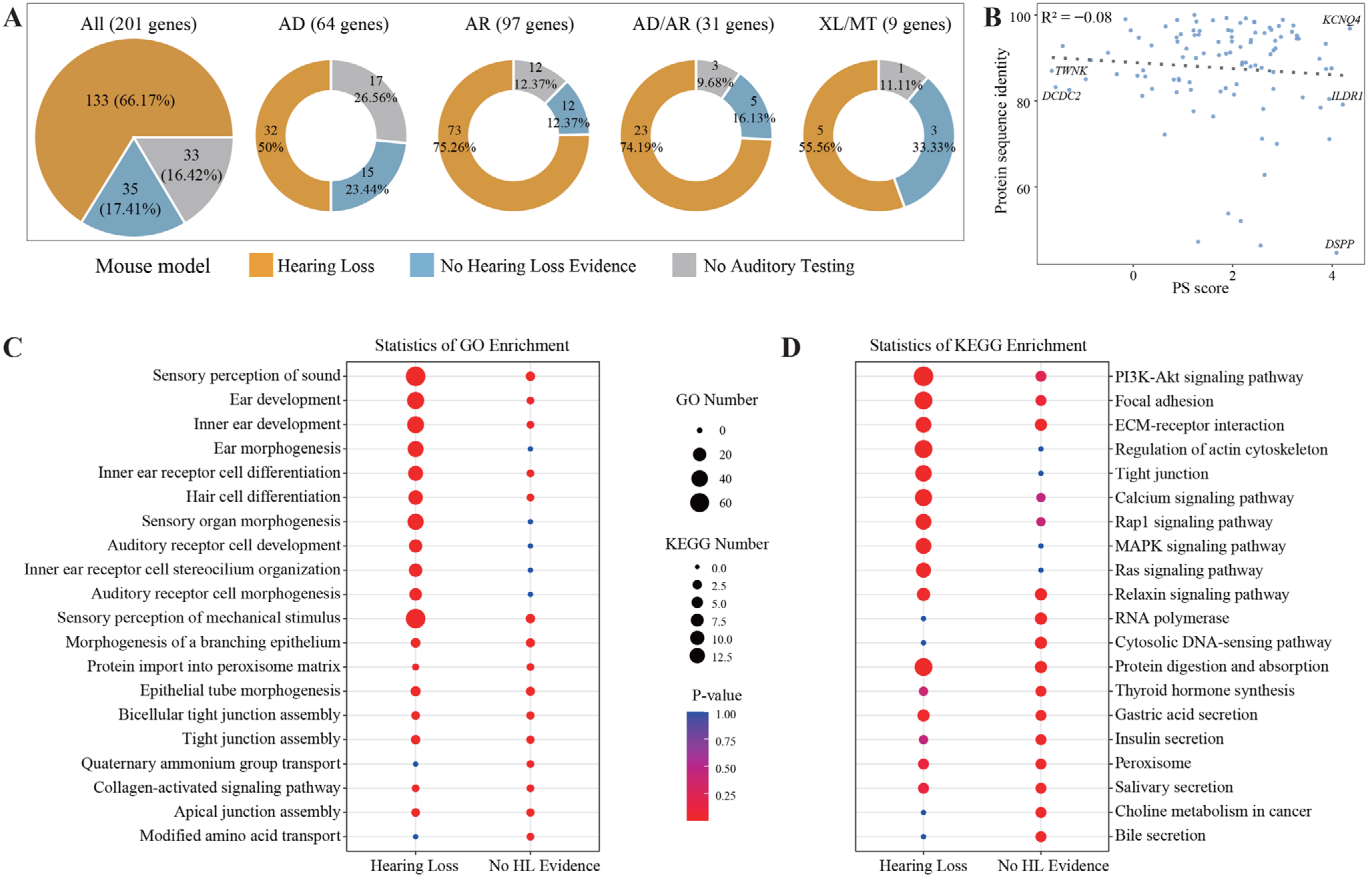
Comparison of human and mouse HL phenotypes. A) Assessing the hearing phenotypes of 201 human HL genes in mouse models. B) No correlation is observed between the phenotypic similarity score and the protein sequence homology. C) GO enrichment analysis on HL and NH gene groups. The top 10 enriched GO terms for each group were selected for display. D) KEGG pathway enrichment analysis on HL and NH gene groups. The top 10 enriched pathways for each group were selected for display.

Phenotypic analysis utilizing model organisms, predominantly mice, is crucial for identifying disease genes and elucidating underlying mechanisms. Nevertheless, phenotypes associated with homologous genes often exhibit substantial differences between humans and mice.^[^ ^54^, ^55^, ^56^ ^]^ By reviewing the mouse phenotype database and literature, we categorized mouse phenotypes into three groups: “HL”, “No HL Evidence,” and “No Auditory Testing”. The classification of mouse phenotypes and the related annotations, along with references, can be found in Table S5 (Supporting Information). Among the 201 human HL genes in GDC, only 133 exhibit hearing abnormalities in at least one genotype of the mouse model, while 35 have available genotypes tested without showing a hearing loss phenotype, and 33 lack relevant auditory testing data (**Figure** **6**A). Notably, mouse models showed greater alignment for genes associated with AR inheritance, while discrepancies in HL phenotypes were observed in 24 out of 97 AR genes, 32 out of 64 AD genes, 8 out of 31 AR/AD genes, and 3 out of 8 XL genes. We introduced a phenotype similarity (PS) score to systematically quantify and compare phenotypes between humans and mice.^[^ ^55^ ^]^ No correlation was observed between the PS score and protein sequence homology (Figure 6B).

Further, we explored the differences in gene ontology (GO) and pathways (KEGG) enrichment between genes with consistent and inconsistent HL phenotypes in humans and mice. The “HL” gene group demonstrated significant enrichment in GO terms related to sensory perception of sound, ear development, and inner ear receptor cell differentiation (Figure 6C). Conversely, the “No HL Evidence” group showed enrichment in functions such as quaternary ammonium group transport and protein import into peroxisome matrix (Figure 6C). Pathway analysis revealed that the “HL” group was significantly enriched in the PI3K‐Akt signaling pathway, ECM‐receptor interaction, and MAPK signaling pathway. In contrast, the “No HL Evidence” group was notably enriched in the RNA polymerase and cytosolic DNA‐sensing pathway (Figure 6D). These variations in function and pathway enrichment likely reflect the divergent roles these genes play in mice, highlighting the varied functions genes may exhibit in different biological contexts.

### 2.6 Expression Features of HL Genes in Human, Mouse, and Rhesus Macaque Cochlea

We compared the expression patterns of HL genes in the cochlea of mouse, human, and rhesus macaque. Single‐cell RNA‐seq data at different developmental stages of the mouse cochlea were from five public repositories (GEO: GSE172110, GSE182202, GSE181454, and GSE202920; and GSA: CRA004814).^[^ ^45^, ^46^, ^47^, ^48^, ^49^ ^]^ For humans, single‐cell RNA‐seq data from intact cochleae of 17‐week postmortem fetuses were sourced (GEO: GSE128505).^[^ ^57^ ^]^ Additionally, bulk RNA‐seq data from adult rhesus macaque cochleae were generated in this study (GSA: CRA020698).

**Figure 7:**
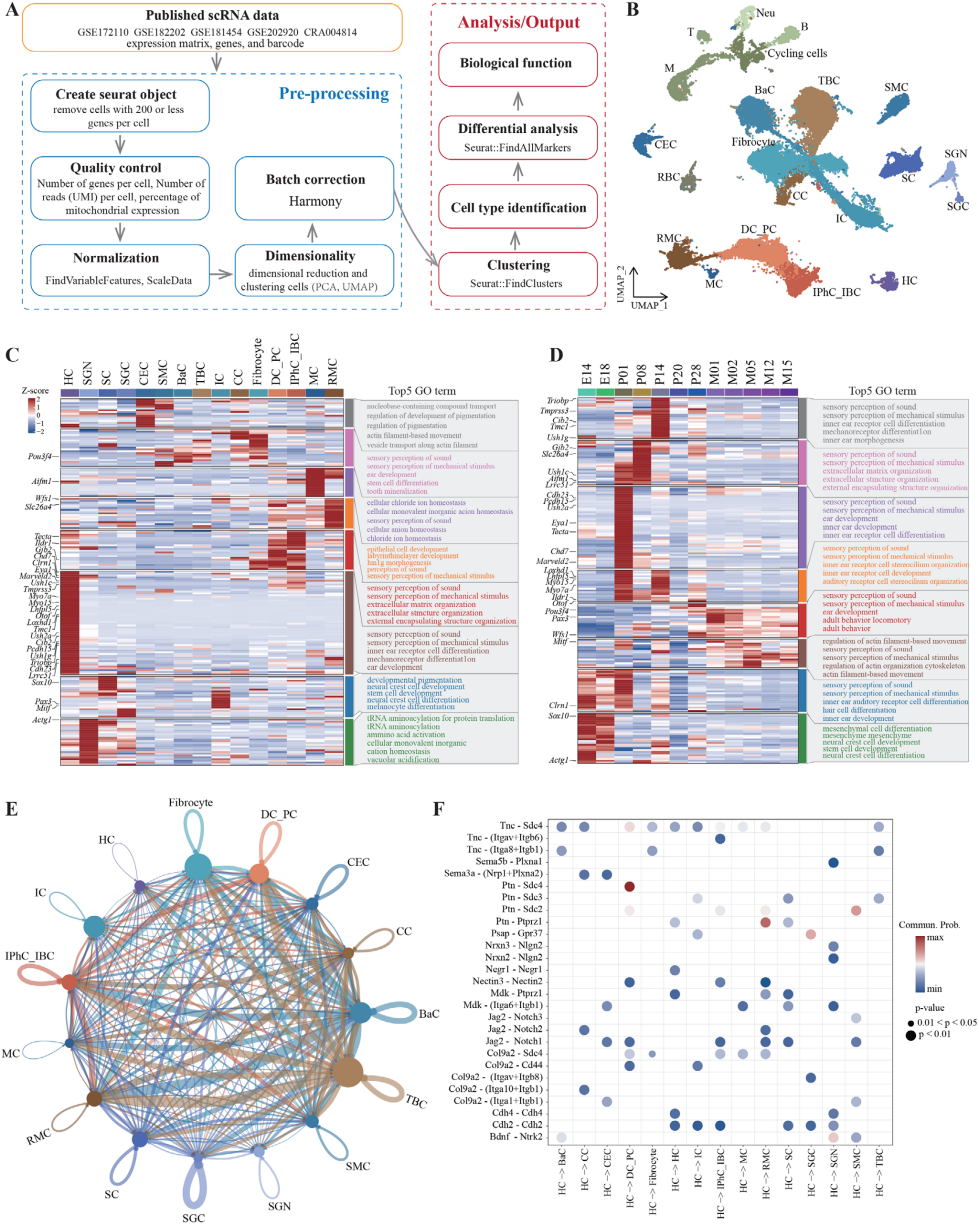
Single‐cell transcriptome landscape of mouse cochlea. A) Analysis process of single‐cell data of mouse cochlea. B) The distribution of different cell types in mouse cochlea. Abbreviations: HC, Hair cell; SGN, Spiral ganglion neuron; SGC, Satellite glial cell; SC, Schwann cell; CEC, Capillary endothelial cell; SMC, Smooth muscle cell; MC, Marginal cell; BaC, Basal cell; TBC, Tympanic border cell; IC, Intermediate cell; CC, Chondrocyte; DC_PC, Deiter cell and pillar cell; IPhC_IBC, Inner phalangeal cell/inner border cell; RMC, cells in Reissner's membrane; T, T cell; B, B cell; M, Macrophage; Neu, Granulocyte/neutrophil; RBC, Red blood cell. C) The expression distribution of the 201 HL genes in different cell types of the mouse cochlea. D) The distribution of the 201 HL genes in different developmental stages of the mouse cochlea. Genes were clustered based on expression patterns in (C,D). The top five enriched GO terms for each cluster are listed. E) Ligand‐receptor pair interactions among all cell types, with the width of edges representing the strength of the communication. F) The identification of ligand‐receptor pairs that may interact with hair cells. Bubble size represents *p* value generated by the permutation test, and the color represents the possibility of interactions. Empty space means the communication probability is zero.

Analysis of single‐cell RNA‐seq data from mouse cochlea at different developmental stages identified 21 major cell types, grouped into 12 clusters (**Figure** **7**A,B, Figure S6A, Supporting Information). These clusters included cells located in and around the organ of Corti, modiolus, Reissner's membrane, stria vascularis, spiral ligament, and various immune cell types. Functional enrichment analysis of the top 50 cell type‐specific marker genes revealed the unique functional roles of these cell types (Figure S6B, Supporting Information). Overall, the expression levels of 201 HL genes were significantly higher in inner ear cells compared to all other genes (Figure S7A, Supporting Information). However, differential expression analysis indicated that HL genes were rarely among the top five most highly expressed genes in hair cells, supporting cells, or other cell types (Figure S7B, Supporting Information).

In mice, gene expression across different cell types enabled the clustering of the 201 HL genes into eight distinct groups. We further highlighted the top 30 genes frequently diagnosed in CDGC patients (Figure 7C). Certain genes, including *Cdh23, Cib2, Clrn1, Eya1, Lhfpl5, Loxhd1, Marveld2, Myo15a, Myo7a, Otof, Pcdh15, Tmc1, Tmprss3, Triobp, Ush1c, Ush1* *g* and *Ush2a*, exhibited high expression levels in hair cells, indicating their crucial roles in inner ear development. Genes such as *Gjb2, Ildr1*, *and Tecta*, predominantly expressed in supporting cells, were vital for maintaining auditory function, as the genetic knockout of *Gjb2* in these cells results in Cx26‐negative hair cell death.^[^ ^58^ ^]^ Other genes, including *Actg1*, *Aifm1*, *Clrn1*, *Pou3f4*, and *Sox10* were expressed in various other cell types, suggesting a broad spectrum of functions.

We also examined gene expression across different developmental stages in mice (Figure 7D). *Actg1*, *Clrn1*, and *Sox10* showed high expression during the embryonic stage, indicating their involvement in early inner ear development. Conversely, *Pax3, Pou3f4, Wfs1*, and *Mitf* showed postnatally high expression, suggesting significant roles in sustaining hearing function during aging.

In the human cochlea, single‐cell RNA‐seq analysis identified 14 major cell types (Figure S7D, Supporting Information), with expression levels of the 201 HL genes significantly higher than those of other genes (Figure S7E, Supporting Information). Similarly, bulk RNA‐seq data from the rhesus macaque cochlea showed consistently higher expression of these 201 HL genes compared to other genes (Figure S7C, Supporting Information). Correlation analyses across species revealed high concordance of overall gene expression levels between humans, rhesus macaques, and mice, with correlation coefficients ranging from 0.718 to 0.752 (Figure S7F, Supporting Information). However, the expression correlation for the 201 HL genes specifically was lower, ranging from 0.533 to 0.605, likely reflecting tissue‐ or cell‐specific expression patterns (Figure S7G, Supporting Information).

We compared gene expression patterns between human and mouse hair cells and supporting cells. The correlation coefficients were notably high, with 0.73 for hair cells and 0.83 for supporting cells. Genes such as *CDH23*, *CIB2*, *CLRN1*, *EYA1*, *LHFPL5*, *LOXHD1*, *MARVELD2*, *MYO15A*, *MYO7A*, *OTOF*, *PCDH15*, *TMC1*, *TMPRSS3*, *TRIOBP*, *USH1C*, *USH1G*, and *USH2A* were highly expressed in both human and mouse hair cells, while *CLDN9*, *COL11A1*, *COL11A2*, *ESRP1*, *EYA1*, *GATA3*, *GJB2*, *GRHL2*, *ILDR1*, *MYH14*, *OTOG*, *OTOGL*, *P2RX2* and *TECTA* were consistently expressed in supporting cells across both species. In contrast, genes such as *AFG2B*, *AIFM1*, *ALMS1*, *ATP1A3*, *BDP1*, *CLPP*, *DCDC2*, *DIABLO*, *GSDME*, *LMX1A*, *MAP1B*, and *TIMM8A* showed differential expression between human and mouse hair cells, while *ACTG1*, *BSND*, *CEACAM16*, *CLIC5*, *ESRRB*, *PEX6*, *S1PR2*, *SOX10*, and *TJP2* displayed differential expression in supporting cells (Figure S8, Supporting Information). Aligning these differentially expressed genes with mouse phenotype data revealed that most genes highly expressed in hair cells or supporting cells were associated with hearing impairment phenotypes in mouse models. However, many genes with differential expression between human and mouse hair cells and supporting cells lacked reported hearing impairment phenotypes in mouse models (Figures S8 and Figure S9A, Supporting Information). We examined the expression correlation of the HL genes between humans and mice, stratified by the cell type and the presence or absence of reported hearing loss phenotypes. The results demonstrated that the “HL” gene group exhibited stronger correlations compared to the “No HL Evidence” group in the hair cells, supporting cells, and Schwann cells (Figure S9B, Supporting Information).

To investigate the specificity of cell‐cell communication within the mouse cochlea, the CellChat R package was employed to predict ligand‐receptor interactions based on scRNA‐seq data. An integrated cell communication network was constructed to assess the interaction strengths among various cell groups. Notably, tympanic border cells and basal cells emerged as the primary contributors to the communication network, highlighting their pivotal roles in intercellular coordination within cochlear tissues (Figure 7E). Given the critical role of hair cells in auditory processes, we further focused on the ligand‐receptor pairs mediating interactions between hair cells and other cell types. The analysis revealed the predominance of the Ptn‐Scd4 communication pathway (Commun. Prob. = 0.37) in hair cell communication with Deiters’ and pillar cells and the Ptn‐Ptprz1 pathway (Prob. = 0.34) in interactions between hair cells and Reissner's membrane cells. Furthermore, the Bdnf‐Ntrk2 pathway (Commun. Prob. = 0.28) exhibited significant activity in hair cell interactions with the spiral ganglion, aligning with findings reported by Ma et al.^[^ ^59^ ^]^ (Figure 7F). These results underscore the significance of potential ligand‐receptor networks, particularly the Ptn‐Scd4, Ptn‐Ptprz1, and Bdnf‐Ntrk2 communication pathways, which may play a crucial role in maintaining auditory function.

### 2.7 Identification of Candidate HL Genes by Integrating Expression and Function Datasets

**Figure 8:**
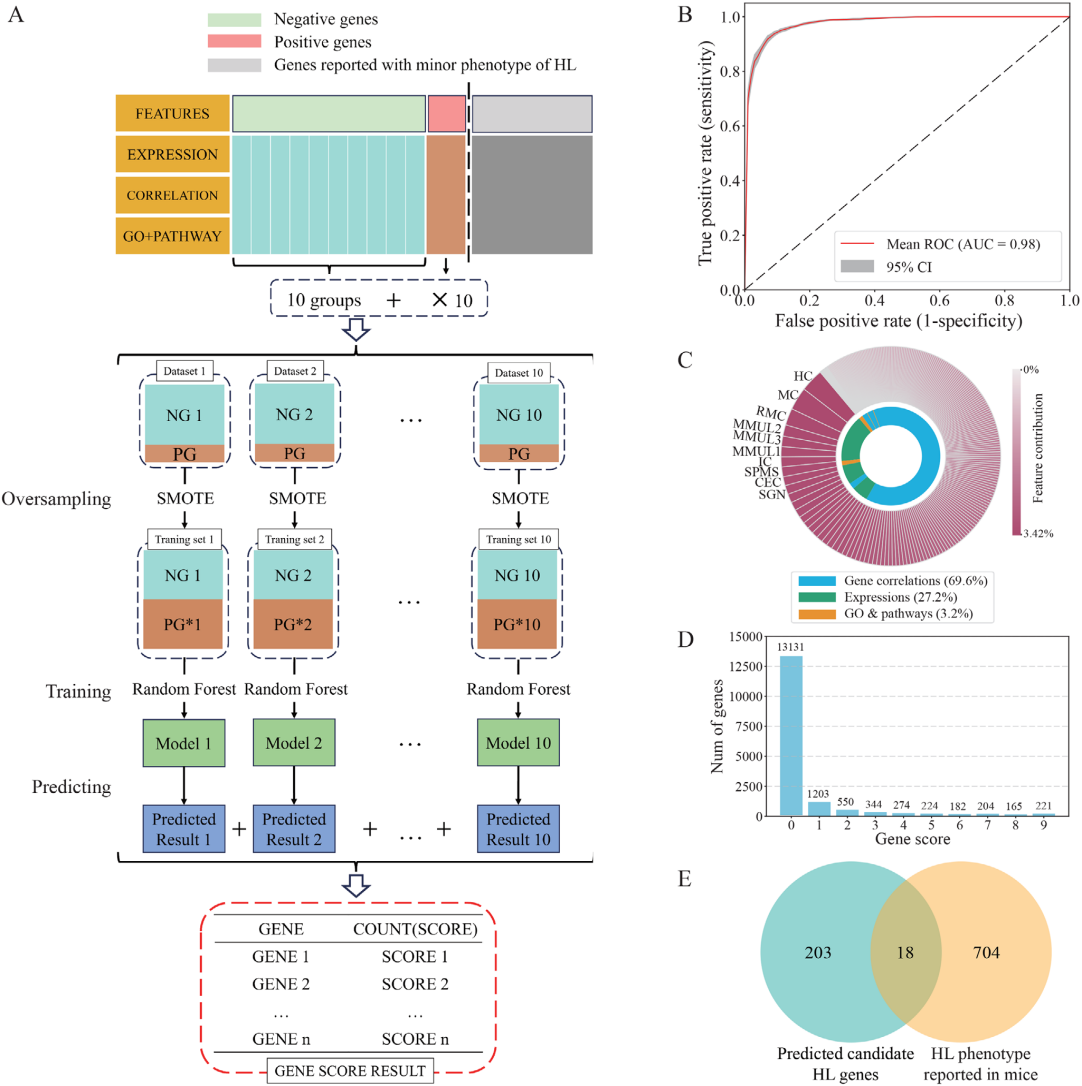
Candidate HL genes discovery via synthetic minority oversampling (SMOTE) Random Forest Model. A) Workflow for gene classification and prediction model. SMOTE method was used for oversampling to create training sets. Abbreviations: NG, HL‐negative genes; PG, HL‐positive genes. B) Mean ROC Curve with 95% Confidence Interval (CI) for 10 SMOTE‐Random Forest classifiers. The red line represents the mean ROC curve. The shaded grey region denotes the 95% CI. C) Feature contribution to the SMOTE‐Random Forest model. The outer ring shows the importance of each feature. The top 10 features are labeled with text. The inner ring categorizes the features into three groups: gene correlation, GO & pathways, and expression. D) Distribution of gene scores. The x‐axis represents the gene scores for HL. The y‐axis indicates the number of genes in each gene score category. E) Overlap between genes with a high score of 9 and genes showing HL phenotype in mice.

Utilizing comprehensive expression and functional annotation datasets incorporated by the GDC, we explored novel candidate genes for HL employing a supervised machine learning approach (**Figure** **8**A). Our analysis incorporated the expression levels of genes across 15 cochlear cell types derived from mouse single‐cell RNA‐seq data and three bulk RNA‐seq experiment results on rhesus macaque cochlea. Additionally, we computed the expression correlations among all genes, utilizing the 201 coefficients correlated with HL genes as features. Along with 30 pathway annotations and 30 GO annotations, it constitutes a total of 275 features to be fed into the subsequent model (Table S6, Supporting Information).

By excluding genes without expression information, the dataset started with 18771 genes. To construct the training set, we initially selected 201 HL‐associated genes for the positive dataset, while the remaining genes constituted the negative dataset. To improve phenotype association accuracy, we excluded four genes from the positive dataset that had been refuted by ClinGen and removed 2081 genes from the negative dataset that were listed in the HPO as having HL as a minor symptom. This refinement yielded a dataset of 197 HL‐positive genes and 16493 HL‐negative genes. Given the significant data imbalance, we implemented the SMOTE‐Random Forest classifier. We randomly divided the 16493 HL negative genes into ten groups, each paired with the 197 HL positive genes to form ten distinct datasets. We then trained these datasets and evaluated them using a split of 0.2 for the test set and 0.8 for the training set. All ten models demonstrated high‐performance metrics (Figure 8B), achieving precision scores ranging from 0.90 to 0.95 (mean of 0.93), recall scores from 0.87 to 0.95 (mean of 0.925), and F1 scores from 0.91 to 0.94 (mean of 0.924). The models also provided insights into feature importance. Among the top 10 important features, the majority (9 out of 10) were related to expressions, including gene expression data in six mouse cochlea cell types and three rhesus macaque cochlea samples, with the only exception being the GO term of sensory perception of mechanical stimulus. Although expression correlations with the known 197 HL genes were not ranked among the top features, they accounted for a significant proportion of the overall feature contribution compared to the other two categories (Figure 8C; Figure S10, Supporting Information).

We performed ten rounds of predictions on HL‐negative genes not included in the corresponding training datasets using the models, identifying 221 genes consistently predicted as positive in all instances (Figure 8D). Among these, 18 genes exhibited HL phenotypes in mice (Figure 8E and Table S7, Supporting Information). Further analysis of patient data from the CDGC cohort and experimental validations pinpointed *TBX2* as a novel HL genes.^[^ ^60^ ^]^

## 3 Discussion

By integrating extensive data from numerous public repositories and the HL patients from the CDGC cohort, GDC substantially advanced the understanding of the genetic landscape of hearing loss. Analyses of the GDC dataset refined the evidence for interpreting variant pathogenicity in specific genes and identified six novel mutational hotspots within key HL genes, highlighting the dynamic nature of genomic regions linked to auditory dysfunction. These insights significantly enrich our diagnostic toolkit, enabling the precise identification of pathogenic variants that can be directly implemented in clinical settings to improve both diagnostic accuracy and patient outcomes. Moreover, further exploration of the GDC dataset through comparative phenotypic analysis highlighted significant disparities between human and mouse models. Detailed examination of gene expression in the cochleae of both mice and rhesus macaques illuminated the conservation and divergence of genes essential for auditory function and development. Leveraging in‐depth data on gene expression, function, and phenotypes, the application of the machine learning approach identified 18 candidate HL genes. Notably, among these candidates, *TBX2* has been validated as a novel HL gene using the patient data from the CDGC cohort, representing a landmark advancement in understanding HL genetics.^[^ ^60^ ^]^

The rapid advancement of DNA sequencing technologies has markedly improved our ability to pinpoint genes and mutations associated with diseases. However, this progress also presents a significant challenge in accurately interpreting the pathogenicity of numerous rare variants. This issue is also evident in the GDC, where up to 95.2% of variants are either of uncertain significance or remain unclassified, highlighting the need for comprehensive integration of clinical, genetic, and molecular functional data to bridge these gaps. In response, the ACMG/AMP jointly developed guidelines to standardize genetic diagnostic procedures, incorporating up to 28 lines of evidence to facilitate variant interpretation and classification.^[^ ^61^ ^]^ This initiative has markedly enhanced the consistency, reproducibility, and transparency of genetic diagnosis. Adapting these guidelines to accommodate the extensive diversity across diseases and genes remains critical for meeting the intricate demands of clinical practice and research. To this end, specific evidence rules, such as PVS1,^[^ ^62^ ^]^ PP3,^[^ ^63^ ^]^ BP4,^[^ ^64^ ^]^ and PS4,^[^ ^65^ ^]^ along with specific guidelines for particular disorders like inherited cardiomyopathy,^[^ ^66^ ^]^ cerebral creatine deficiency syndromes,^[^ ^67^ ^]^ and genetic HL,^[^ ^68^ ^]^ have been continuously optimized and refined over recent years. Presently, the GDC covers 17 out of 24 lines of evidence applicable to genetic HL as outlined by HL‐ACMG,^[^ ^68^ ^]^ providing substantial support for clinical genetic diagnosis.

More importantly, the GDC provided new insights that enhance the application of the ACMG/AMP guidelines for clinical genetic diagnosis. The PM1 rule, defined as the significant enrichment of pathogenic missense variants in a mutational hotspot and/or critical and well‐established functional domain, represents an important line of pathogenic evidence. However, the practical implementation of the PM1 was constrained by a scarcity of studies on the identification of such hotspots. In the context of genetic HL, only collagen genes and the *KCNQ4* gene were previously reported to have mutational hotspots amenable to PM1.^[^ ^53^, ^68^ ^]^ In this study, we systematically mapped the distribution of pathogenic missense variants at the amino acid level across 201 HL genes, identifying 12 genes with hotspots enriched for pathogenic variants. According to Tavtigian et al.,^[^ ^52^ ^]^ the positive likelihood ratio of eight enriched genes, including six reported for the first time in this study, exceeds thresholds of supporting or moderate strength level, thus broadening the applicability of this rule. Notably, among all 12 transcription factor genes examined, 10 exhibited enrichments in DNA binding domains, highlighting the critical amino acids within these domains that impact transcriptional efficacy. By pinpointing these hotspots, this discovery provides valuable insights into the genetic landscape of deafness and establishes a foundation for subsequent studies on the functional implications of HL genes.

Another specific rule addressed by this study focused on NMD‐escape premature termination codon variants, which present challenges in genetic diagnosis due to their varied impacts on protein function. A key determinant of NMD efficiency is the position of PTVs, encapsulated by the 50 nucleotide (50 nt) boundary rule: variants occurring more than 50 to 55 nucleotides upstream of the last exon junction are targeted for degradation.^[^ ^69^, ^70^ ^]^ Conversely, PTVs located downstream of this boundary are considered NMD‐escape and are subject to a reduction in the strength of the pathogenic PVS1 rule for genetic diagnostics.^[^ ^62^ ^]^ The pathogenic mechanisms of NMD‐escape PTVs may include the production of a truncated protein leading to a LoF effect, or alternatively, the truncated protein may exert a dominant negative or a GoF effect, with impacts varying according to the specific gene and variant.^[^ ^71^ ^]^ This study identified a significant number of predicted NMD‐escape PTV variants in multiple HL genes, including *MARVELD2*, *MYH9*, and *MYH14*. The results suggest that subsequent identification of NMD‐escape PTVs in these genes is not subject to reduction of evidence strength, it also revealed the function implication that the C‐terminal amino acids of these proteins are essential for their functionality, and disruption at these sites may compromise the entire protein function.

Constructing animal models that accurately simulate human phenotypes is essential for validating disease genes and exploring underlying mechanisms. The mouse model has been widely used in hearing research due to its short generation time and the structural similarity of its cochlea to that of humans, which allows for effective dissection and imaging.^[^ ^72^ ^]^ Despite this, discrepancies between human and mouse phenotypes are not uncommon. Among the 201 human HL genes, 168 had corresponding mouse models that underwent auditory testing, while 35 genes (21%) still lacked evidence of a hearing loss phenotype in mice to date. Interestingly, these discrepancies appear minimally related to the sequence homology of the corresponding proteins or gene expression levels, suggesting that they may stem from a higher order of genomic evolution and organization.^[^ ^54^ ^]^ Comparative genomics and transcriptomics studies have indicated that the phenotypic differences between species are partly due to variations in non‐coding regulatory elements and transcriptome profiles, rather than differences in protein‐coding sequences.^[^ ^55^, ^73^ ^]^ Additionally, the timing of gene expression can significantly influence phenotypic outcomes in mice. For instance, the downregulation of Cx26 at different postnatal stages has varying effects on hearing.^[^ ^74^ ^]^ Furthermore, the genetic background of different mouse strains significantly affects phenotypic expression, as exemplified by the *Atp6v1b1* gene, which failed to induce HL when knocked out in the C57 background but succeeded in the MRL/MpJ background.^[^ ^75^ ^]^ Genetic redundancy, where a gene deletion or mutation is compensated for by other genes, may also influence phenotypic variations.^[^ ^76^ ^]^ To overcome these challenges, strategies such as constructing humanized mouse models, knocking out additional genes within the same family, selecting different mouse strains, and incorporating environmental factors have been proposed. Successful humanization of mouse models for deafness genes, like *MYO6* ^[^ ^77^ ^]^ and *COCH*,^[^ ^78^ ^]^ has yielded critical insights into the molecular mechanisms of hearing diseases.

Research shows that a significant proportion of orthologous protein‐coding genes maintain conserved expression patterns between humans and mice,^[^ ^73^ ^]^ a result that aligns with our findings of strong correlation in gene expression levels between mouse and rhesus macaque cochlear tissues (Figure S7F, Supporting Information). Leveraging these insights, we integrated gene expression data from both species, incorporating expression correlations, pathways, and functional data into the feature set. Using the SMOTE‐Random Forest Classifier to manage oversampled datasets, we developed a scoring model to identify candidate genes for HL. The model was primarily driven by gene expression levels across different cell types, indicating potential improvements by including more types of omics data such as proteomics and epigenomics.^[^ ^79^, ^80^ ^]^ Subsequent overlapping of HL phenotype data from mouse models facilitated the filtering of candidate genes. Through genetic analyses within the CDGC cohort and subsequent experimental validation, we confirmed one novel HL gene *TBX2*.^[^ ^60^ ^]^ These results underscored the value of extensive, multi‐layered genetic and genomic data in the GDC, demonstrating how the use of such data can aid in uncovering novel disease genes and deepen our understanding of disease mechanisms.

The results of this study should be considered in light of several limitations. A significant proportion of variants within the GDC remain unclassified or of uncertain significance, which could potentially obscure our understanding of the genetic landscape of HL. Moreover, the GDC exhibited only a limited number of pathogenic non‐coding variants across a handful of genes. The analysis of non‐coding variants is notably challenging due to their intricate regulatory roles and the absence of clear clinical correlations. To improve the accuracy of interpreting both coding and non‐coding variants, it is crucial to integrate more comprehensive genetic data from a broad range of ethnic groups and to innovate in the development of new high‐throughput functional assays and analytic algorithms. Another critical obstacle is the difficulty in obtaining human inner ear tissue samples, which restricts the generation of comprehensive epigenetic and expression data that are essential for a thorough understanding of the molecular mechanisms underlying auditory functions and deafness. Addressing these limitations is crucial for future research efforts aimed at advancing our capabilities to explore and interpret auditory‐related issues and develop effective therapeutic strategies for deafness.

In conclusion, GDC represents a valuable endeavor, serving as a unified and enriched information repository that captures data and knowledge to specifically advance research and clinical practices in audiology and hearing‐related conditions. Future enhancements of the GDC will focus on expanding coverage to additional HL‐related genes, incorporating epigenetic regulatory information of cochlea, and developing the knowledge graph through literature mining, all of which will further solidify its role as an invaluable resource for the research community.

## 4 Experimental Section

### CDGC Cohort Study

The CDGC project was a China‐wide cooperative network of medical genetics research on hearing loss and related disorders. The study aims to elucidate the role of genetic variation in the pathogenesis of HL by bringing together the increasing genetic information and high‐quality clinical data, as well as analysis expertise, to discover the basis for the prevention and treatment of HL, and to exploit the new knowledge to reduce the burden of disease. Since 2013, the CDGC project has involved 22125 cases and 7254 controls across mainland China. The exclusion criteria were: 1) conductive HL (e.g., HL secondary to otitis media, chronic myringitis, perforated eardrum, and tympanosclerosis); 2) presbycusis (age of onset > 40 years); 3) unilateral HL without a family history; and 4) mild HL without a family history. The involved cases went through three‐stage genetic testing using SNPscan, CDGC‐HL panel, and whole genome sequencing (WGS).^[^ ^81^ ^]^

Targeted genetic analysis was performed on 201 HL‐related genes reported by 2022. NSHL genes were curated by systematically screening relevant genes from authoritative sources, including Hereditary Hearing Loss Homepage,^[^ ^8^ ^]^ ClinGen,^[^ ^43^ ^]^ OMIM,^[^ ^42^ ^]^ and publications. For SHL genes, only syndromes where hearing loss was a primary phenotype were included. These syndromes were either cataloged in the Hereditary Hearing Loss Homepage or determined based on our extensive clinical experience. Genes associated with these syndromes were subsequently retrieved from public databases and literature. Variant interpretation and genetic diagnosis were performed on up to 201 HL genes based on the set of ACMG/AMP guidelines and the updates,^[^ ^61^, ^62^, ^63^, ^68^, ^82^, ^83^ ^]^ in the context of patient phenotype, onset age, HL severity, other otologic testing records, family history, and medication history. Putative diagnostic variants were Sanger sequenced for validation. All variants identified in the CDGC cohort in 201 HL genes were included in the GDC, with information on pathogenicity classifications and allele frequencies.

### Data Integration and Development of GDC

GDC integrates genetic and phenotypic information from 51 public databases. Each gene was annotated with 11 aspects of information from 29 databases, including summary information, phenotype, and disease correlation, gene expression, subcellular localization, protein‐protein interaction, gene function and pathway, animal model, as well as gene‐drug interaction (Table S1, Supporting Information). Variants in GDC were incorporated from multiple data sources in addition to CDGC, including ClinVar, DVD, HGMD, gnomAD, and ChinaMAP. A total of 27 databases provide variant annotations, including functional consequence and impact, allele frequencies in different populations, pathogenicity classification, computational prediction scores, correlated diseases or phenotypes, and linked publications. Variant Effect Predictor (VEP) was employed to provide comprehensive annotations for each variant.^[^ ^84^ ^]^ All data were standardized and stored in the MongoDB database. The GDC website (http://gdcdb.net/) was developed using the Python‐Flask, Nginx, and React JavaScript frameworks, with UI and components built using Antd Design. The web interface of GDC provides user‐friendly searching functions that were equipped to automatically recognize four types of key terms: gene symbols, genomic coordinates of variants, phenotype terms, and disease names. In addition, GDC offers downloadable datasets in VCF/BED/GFF formats for offline annotation.

### Pathogenic Missense Variant Density Estimation and Likelihood Ratio Calculation

P/LP missense variants from CDGC, ClinVar, DVD, and HGMD, and non‐P/LP missense variants from the gnomAD population with maximum MAF < 0.0001 were respectively utilized to estimate the distribution density in terms of the amino acid location. Gaussian kernel density from the NumPy package was used to estimate the density of missense variants and to draw the density map for each gene. The enrichment region was determined based on the location of missense variants and protein function domains retrieved from InterPro. The bootLR^[^ ^85^ ^]^ and epiR^[^ ^86^ ^]^ packages were used to calculate the likelihood ratio (LR), odds ratio (OR), and Fisher's exact *p*‐value of enrichment for each region, employing the following formulas:

(1) $$\begin{array}{c} LR^{+}=\frac{\left(\#P/LP\;variants\;in\;the\;domain\right)/\left(\#P/LP\;variants\;in\;the\;gene\right)}{1-\left(\#NonP\;variants\;outside\;the\;domain\right)/\left(\#NonP\;variants\;in\;the\;gene\right)} \end{array}$$

(2) $$\mathrm{OR}\;=\frac{\left(\#P/LP\;variants\;in\;the\;domain\right)*\left(\#NonP\;variants\;outside\;the\;domain\right)}{\left(\#P/LP\;variants\;outside\;the\;domain\right)*\left(\#NonP\;variants\;in\;the\;domain\right)}$$

where *#* is the number of variants and NonP indicates non‐pathogenic variants.

### Single‐Cell Expression Analysis of the Human and Mouse Cochlea

Single‐cell transcriptomic data of cochlea were retrieved from GEO (Human Accession ID: GSE135913; Mouse Accession ID: GSE172110, GSE182202, GSE181454, and GSE202920; GSA: CRA004814). The human cochlear data used were obtained from postmortem fetuses at a gestational age of 17 weeks. Data analysis and normalization were performed using the R package Seurat v4.2.1.^[^ ^87^ ^]^ For mouse cochlear single‐cell data, expression profiles were analyzed at different developmental stages: E14, E18, P1, P8, P14, P20, P28, M1, M2, M5, M12, and M15. The matrix of read‐count data of each cell for each gene was loaded individually and converted to Seurat objects. To get high‐quality cells, only cells with more than 200 genes and less than 5000 genes were detected, and less than 10% mitochondrial genes were used for subsequent analysis. Gene expression was normalized based on the “NormalizeData” function with default parameters. The Harmony R package was used to adjust batch effects between different samples and integrate the gene expression matrices of all samples.^[^ ^88^ ^]^ The integrated dataset was subsequently used for downstream dimensionality reduction and clustering analyses. Total cell clustering was performed by the “FindClusters” function to define cell identity. Dimensionality reduction was performed with the “RunUMAP” function. Marker genes for each cluster were determined with the Wilcoxon rank‐sum test by the “FindAllMarkers” function. Only genes with a fold change greater than 2 and Benjamini‐Hochberg adjusted *p*‐value less than 0.05 were considered to indicate statistical significance. Cell types were identified based on the expression of classic marker genes. The “clusterProfiler” package^[^ ^89^ ^]^ (version 4.9.5) was utilized to conduct GO and Pathway analysis.

### Cell–Cell Communication Analysis

Cell–cell communication analysis based on the expression of known ligand‐receptor pairs in different cell types was performed using the CellChat R package.^[^ ^90^ ^]^ The overall interaction number and interaction strength in differential cell types were quantified. Finally, the “netVisual_circle” and “netVisual_bubble” functions were applied to visualize communication probabilities by ligand‐receptor pairs in different directions. Significant interaction pairs, with a *p*‐value below 0.05, were considered important.

### Candidate HL Gene Prediction Using SMOTE‐Random Forest Classifier

To identify candidate HL genes, the machine learning approach was utilized on data integrated by GDC. The initial dataset comprised genes characterized by 275 features, including expression levels in mouse and rhesus macaque cochlea, expression correlation with confirmed HL genes, GO terms, and pathways. Confirmed HL genes were labeled positive, while those not listed in the HPO as having HL as a minor symptom were labeled negative. The negative genes were randomly divided into ten subsets, each combined with the positive genes to form ten training datasets. Given the imbalance in the ratio of positive to negative labels (≈9:1) in each dataset, the Synthetic Minority Over‐sampling Technique (SMOTE) was applied to oversample the positive class. A Random Forest Classifier was employed to generate ten models, each predicting the labels of genes not included in its training dataset, yielding nine prediction outcomes for each negative gene. To synthesize these results, the predictions from the ten models were aggregated, counting the number of times each gene was predicted as positive across all models (ranging from 0 to 9 times). This count served as a score indicating the likelihood of a gene being associated with HL, with higher scores suggesting a greater probability. Furthermore, a mean decrease impurity analysis was conducted for the 275 features in each model, yielding an importance score for each feature. A higher feature score signified a more significant influence on the model, indicating a pivotal role in the classification task.

### RNA‐Seq of Rhesus Macaque Cochlea and Data Analysis

Three cochlear samples were retrieved from two rhesus macaques via dissection. Total RNA was extracted from these samples, and mRNA was subsequently enriched using Oligo(dT) magnetic beads. A fragmentation buffer was added to break the mRNA into short fragments. These fragments served as templates for synthesizing the first strand of cDNA using random hexamer primers. Subsequent steps involved adding buffer, dNTPs, RNase H, and DNA polymerase I to synthesize the second cDNA strand. The cDNA was then purified using the QIAquick PCR Purification Kit (Qiagen) and eluted with EB buffer. End repair processes were followed by the addition of poly(A) tails and the ligation of sequencing adapters. The desired fragment sizes were selected via agarose gel electrophoresis, followed by PCR amplification. The prepared sequencing library was then sequenced on the Illumina HiSeq platform.

Raw reads in FASTQ file format were filtered for quality control using Fastp and FastQC software.^[^ ^91^ ^]^ After confirming the quality, clean reads were mapped to the Macaca mulatta genome using Hisat2 software.^[^ ^92^ ^]^ The mapped reads were quantified using StringTie software.^[^ ^93^ ^]^ Transcripts per million (TPM) normalized the RNA‐Seq data, calculated as the read number of a transcript divided by the total clone count of the sample, then multiplied by 10^6^. Differential gene expression between the samples was determined using the R package EdgeR.^[^ ^94^ ^]^

### Statistical Analysis

All statistical analyses and visualizations were conducted using R (v4.3.2) and Python (v3.9.13). Single‐cell RNA‐seq data were obtained from 13362 human cochlear cells (*n* = 1) and 62842 mouse cochlear cells (*n* = 12), while bulk RNA‐seq data were collected from three rhesus macaque cochleae (*n* = 3). Mean gene expression values were either log2‐transformed or standardized as z‐scores. Pearson's correlation analysis was performed to evaluate the association of gene expression levels across the three species. To quantify relative differences in gene expression between humans and mice, the absolute z‐score values of the two species were subtracted and divided by the larger z‐score value. Statistical significance was defined as *p* < 0.05.

## Conflict of Interest

The authors declare no conflict of interest.

## Author Contributions

H.C., X.W., M.Z., J.G., and W.L. contributed equally to this work. H.C. investigated, performed formal analysis, collected and standardized data, and wrote the original draft. X.W. developed, optimized, and tested the website. M.Z. performed formal analysis, collected and standardized data, and reviewed & edited the writing. J.G. performed formal analysis, collected and standardized data, and reviewed & edited the writing. W.L. and K.P. performed formal analysis and reviewed & edited the writing. J.W. generated the rhesus monkey data. L.W. performed formal analysis. Y.L., J.C., F.B., and H.Y. designed the study, reviewed & edited the writing, supervised the project, administered the project, and acquired funding.

## Supporting information



Supporting Information

Supporting Tables

## Data Availability

The data that support the findings of this study are available from the corresponding author upon reasonable request.
