# Supplementary material for: GDC: Integration of Multi‐Omic and Phenotypic Resources to Unravel the Genetic Pathogenesis of Hearing Loss
Source: Adv Sci (Weinh). 2025 Mar 16;12(29):2408891. doi: 10.1002/advs.202408891 (PMC12362786; doi:10.1002/advs.202408891)
Supplement: Supplementary file 1 — Supporting Information [file ADVS-12-2408891-s001.docx]

Supplementary Materials for

**GDC: Integration of Multi-Omic and Phenotypic Resources to Unravel the Genetic Pathogenesis of Hearing Loss**

*Hui Cheng*^1,2,#^, *Xuegang Wang*^1,2,#^, *Mingjun Zhong*^1,2,#^, *Jia Geng*^1,2,#^, *Wenjian Li*^1,2, #^, *Kanglu Pei*^1,2^, *Jing Wang*^1^*, Lanchen Wang*^1,2^, *Yu Lu*^1,2,*^, *Jing Cheng*^1,2,*^, *Fengxiao Bu*^1,2,*^, *Huijun Yuan*^1,2,*^

*Corresponding author: luyu@wchscu.cn (Y. Lu); chengjing@wchscu.cn (J. Cheng); bufengxiao@wchscu.cn (F. Bu); yuanhj301@wchscu.cn (H. Yuan)

**This PDF file includes:**

Figs. S1 to S10

**Other Supplementary Material for this manuscript includes the following:**

Tables S1 to S7


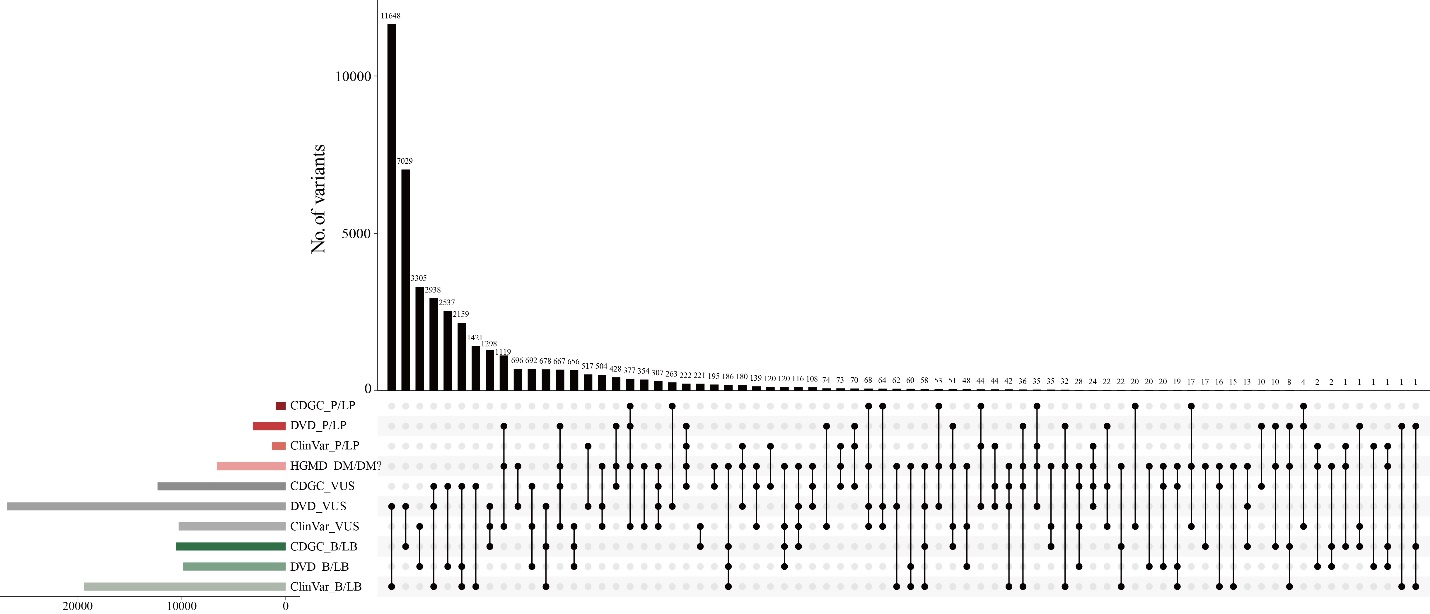


**Supplemental Figure S1:** Variant classification conflicts among CDGC, DVD, ClinVar, and HGMD.


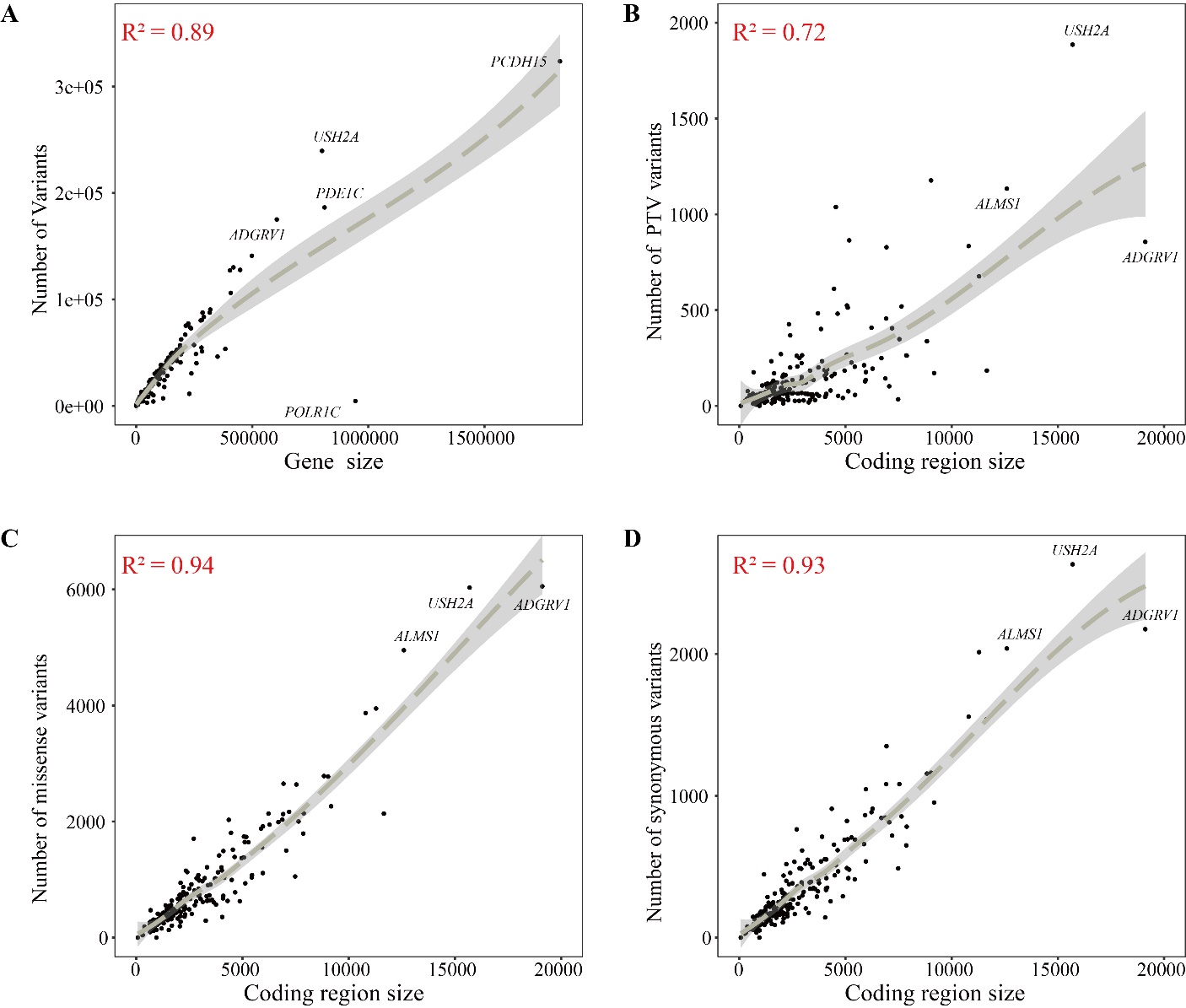


**Supplemental Figure S2:** Correlation between gene length and the number of variants.

(A) Correlation between gene length and the total number of variants. (B) Correlation between coding region length and the number of PTVs. (C) Correlation between coding region length and the number of missense variants. (D) Correlation between coding region length and the number of synonymous variants.


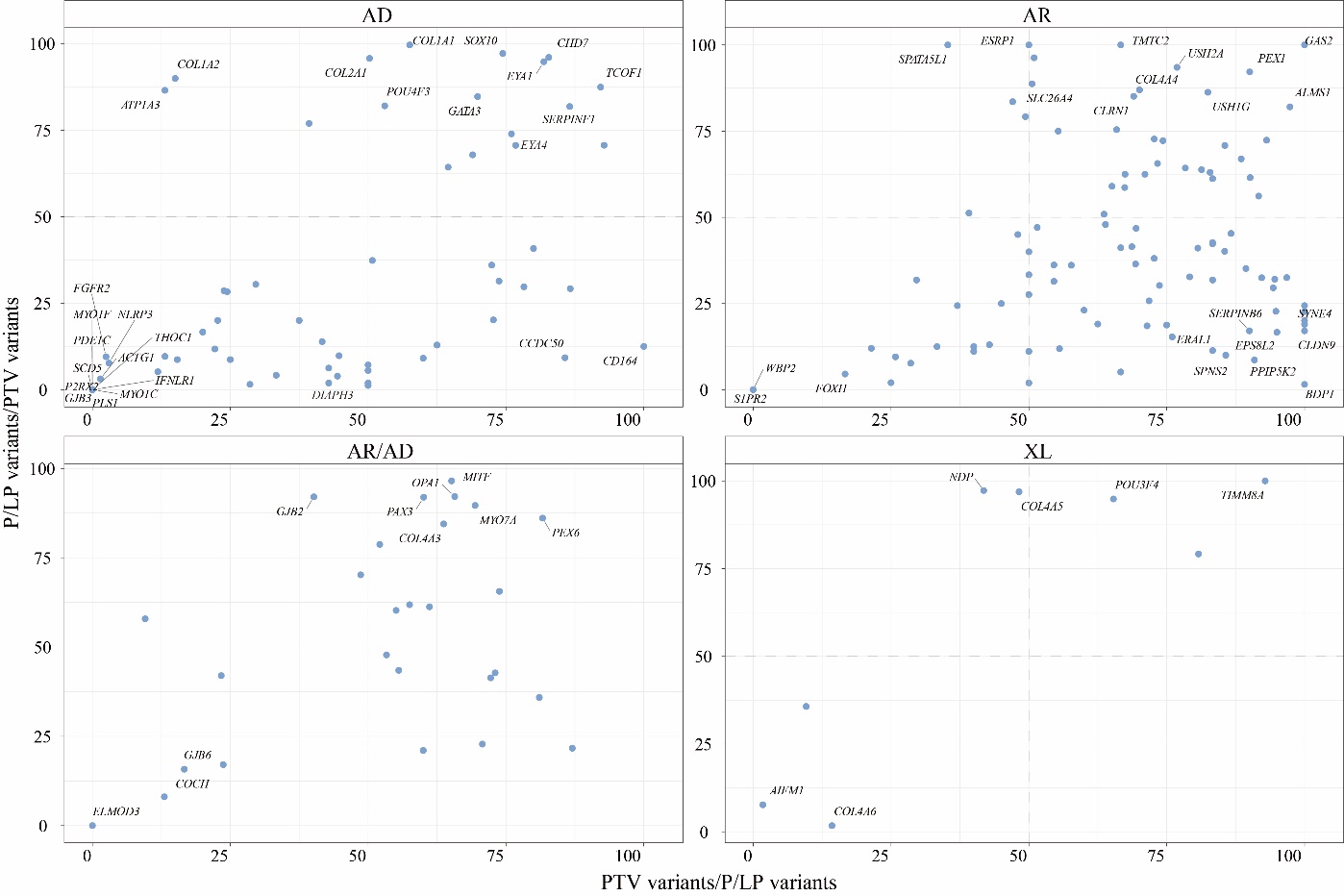


**Supplemental Figure S3:** Proportional distribution of PTVs and P/LP variants.

The X-axis displays the ratio of PTVs within P/LP variants, while the Y-axis shows the ratio of P/LP variants within PTVs. Genes are labeled when the proportion is less than 20% or greater than 80%.


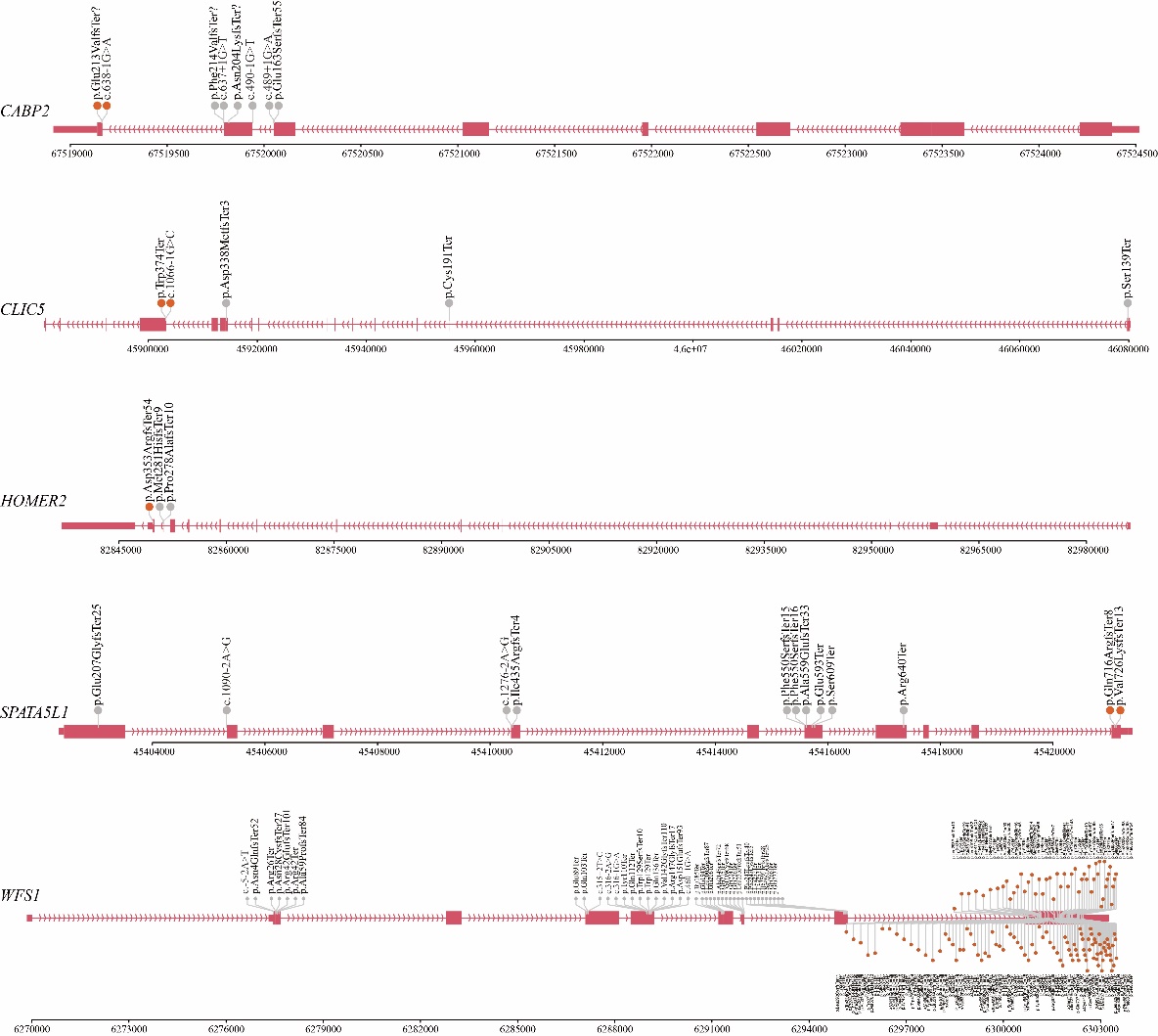


**Supplemental Figure S4:** Distribution of PTVs in genes enriched with NMD-escape PTVs. The orange circle indicates that the variants are within the last exon or the final 50 base pairs of the penultimate exon.


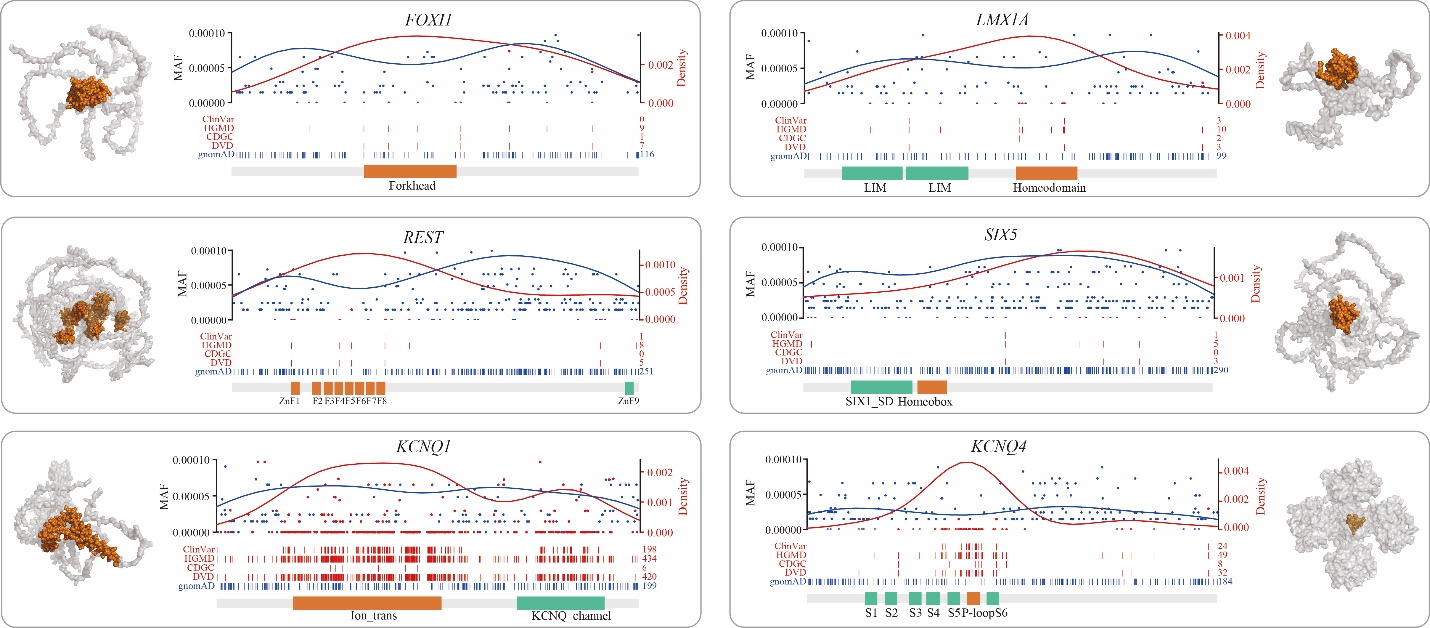


**Supplemental Figure S5:** Identification of hotspots enriched with pathogenic missense variants.

Orange indicates the DNA-binding domains in the gene. Abbreviations: LIM, LIM domain; ZnF, Zinc finger; SIX1_SD, Transcriptional regulator, SIX1, N-terminal SD domain.


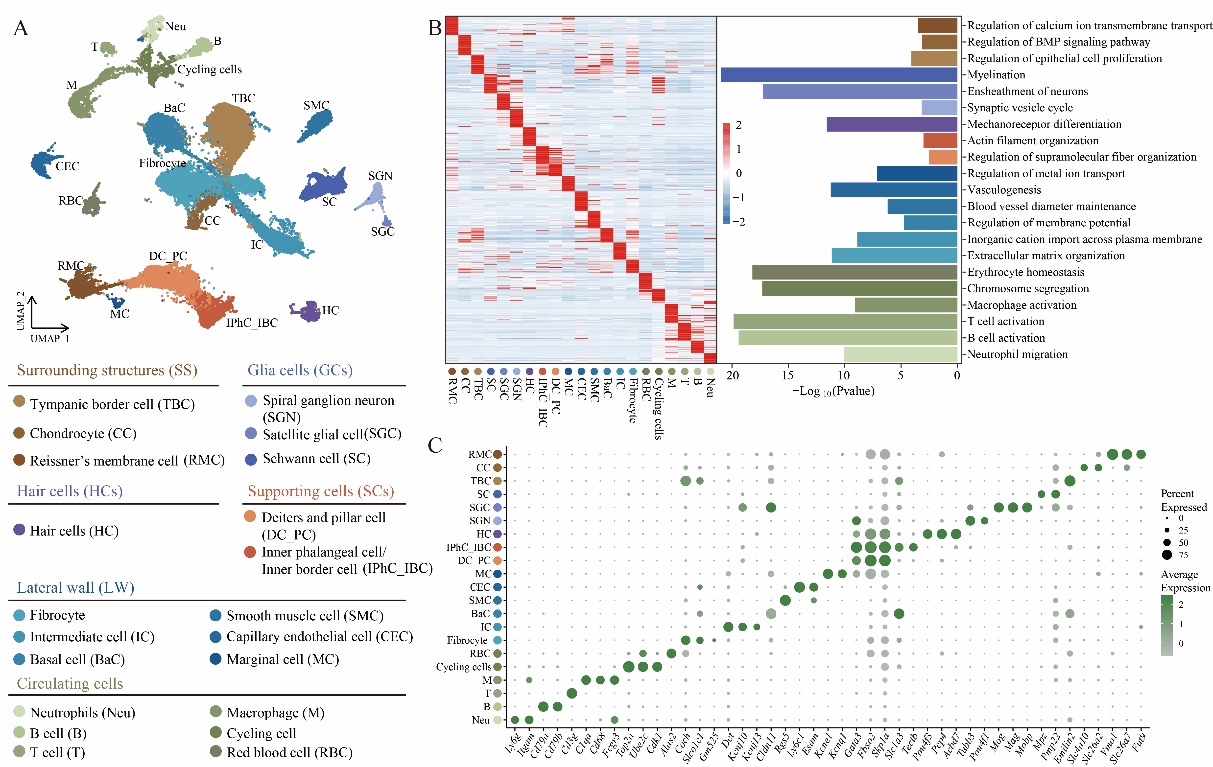


**Supplemental Figure S6:** Establishment of single-cell transcriptome landscape of mouse cochlea.

(A) Distribution of different cell types in cochlea. The annotation of different cell types are: HC, Hair cell; DC_PC, Deiter cell and pillar cell; IPhC_IBC, Inner phalangeal cell/inner border cell; TBC, Tympanic border cell; CC, Chondrocyte; RMC, cells in Reissner’s membrane; SGN, Spiral ganglion neuron; SGC, Satellite glial cell; SC, Schwann cell; IC, Intermediate cell; BaC, Basal cell; CEC, Capillary endothelial cell; SMC, Smooth muscle cell; MC, Marginal cell; T, T cell; B, B cell; M, Macrophage; Neu, Granulocyte/neutrophil; RBC, Red blood cell. (B) Left, heatmap showing row z-score expression signatures of top 50 cell-type-specific genes. Right, representative Gene Ontology (GO) terms for top 50 genes. (C) Expression of canonical cell-type-specific marker genes for different cell types.


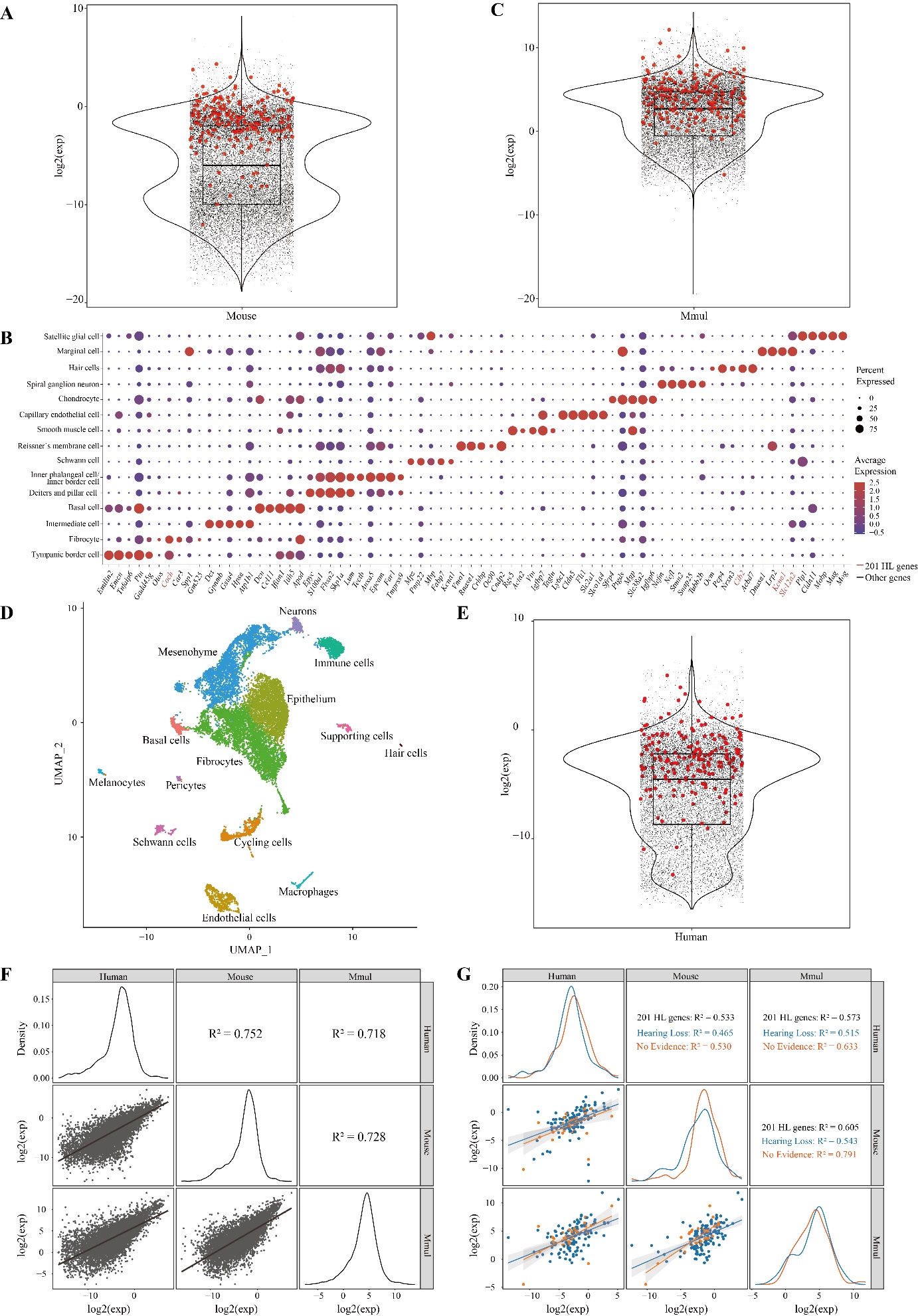


**Supplemental Figure S7:** Gene expression comparison between mouse and rhesus macaque cochlea. (Mouse data: GSE172110, GSE182202, GSE181454, GSE202920, CRA004814; Human data: GSE135913; Rhesus macaque: CRA020698).

(A) Distribution of gene expression in mouse cochlea. Red indicates 201 HL genes. (B) The top 5 highly expressed genes in different cell types of the mouse cochlea. The red-highlighted genes represent the HL genes. (C) Gene expression distribution in cochlea of rhesus monkey. Red indicates 201 HL genes. (D) The distribution of different cell types in human cochlea. (E) Gene expression distribution in cochlea of human. Red indicates 201 HL genes. (F) Expression correlation of all genes in cochlea of human, rhesus macaque and mouse. The scatter plots in the lower corner panels show gene expression correlations among the three species. The diagonal panels show the density distribution of gene expression levels within each species. The upper corner panels present the correlation coefficients across the three species. (G) Expression correlation of HL genes in cochlea of human, rhesus macaque and mouse. The correlation coefficients of 201 genes are presented in black. Genes in the "HL" group are represented in blue, while genes in the "No HL Evidence" group are shown in orange.


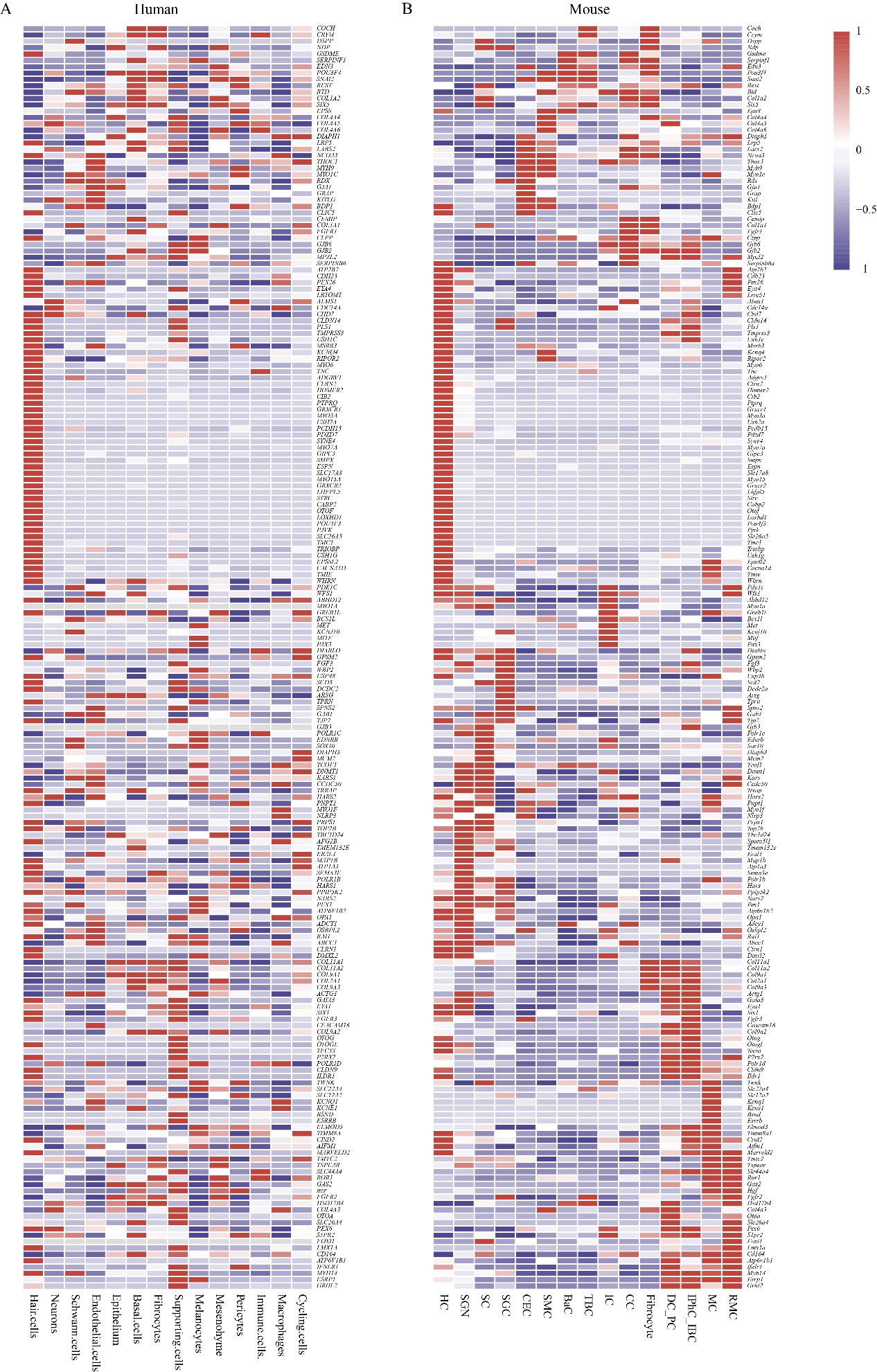


**Supplemental Figure S8:** Gene expression profiles in the human and mouse cochlea.

(A) Expression distribution of the 201 genes in the human cochlea. (B) Expression distribution of the 201 genes in the mouse cochlea. Abbreviations: HC, Hair cell; SGN, Spiral ganglion neuron; SC, Schwann cell; SGC, Satellite glial cell; CEC, Capillary endothelial cell; SMC, Smooth muscle cell; BaC, Basal cell; TBC, Tympanic border cell; IC, Intermediate cell; CC, Chondrocyte; DC_PC, Deiter cell and pillar cell; IPhC_IBC, Inner phalangeal cell/inner border cell; MC, Marginal cell; RMC, cells in Reissner’s membrane.


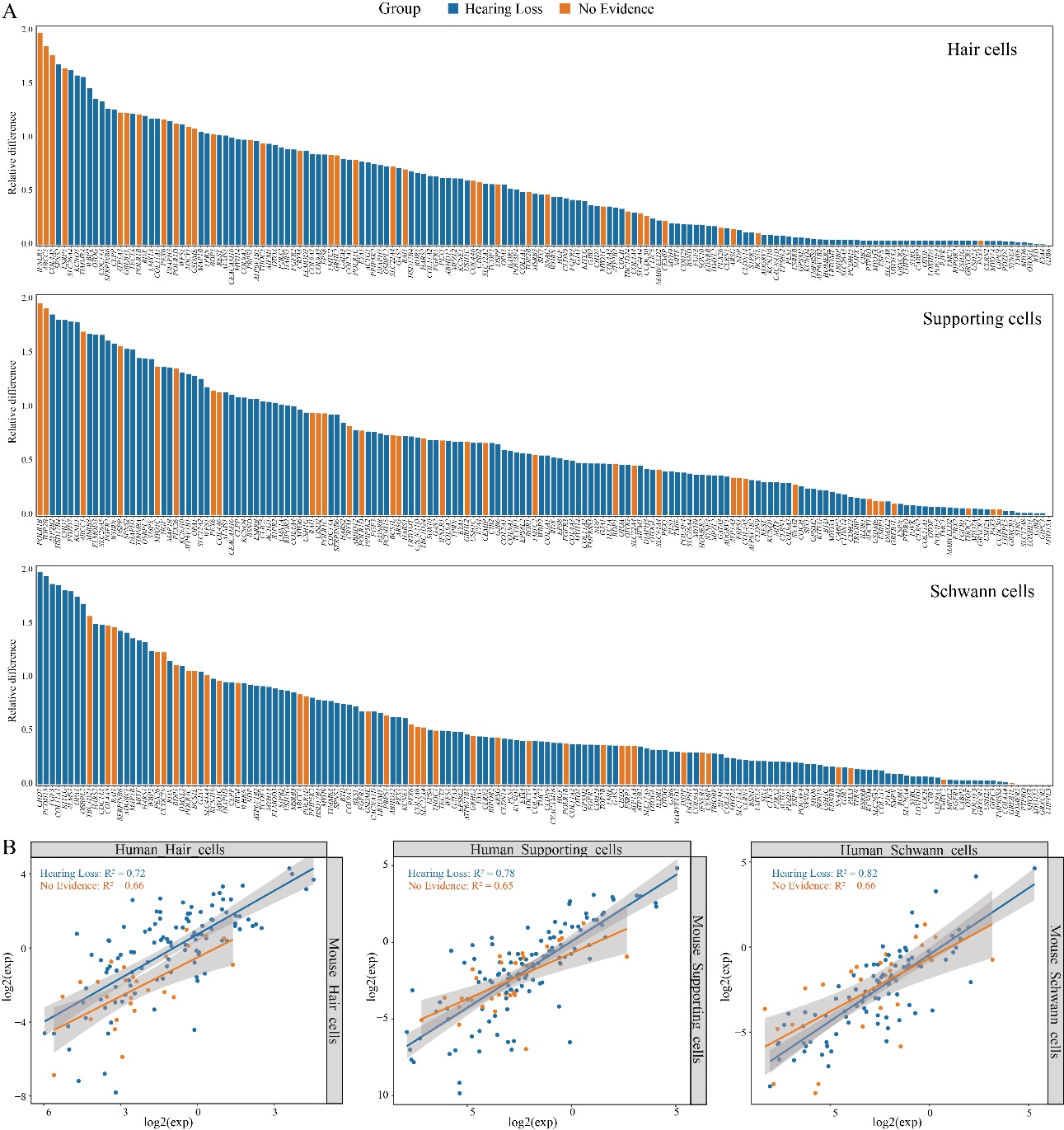


**Supplemental Figure S9:** The correlation of HL genes in hair cells, supporting cells, and Schwann cells in the human and mouse cochlea.

(A) Differential analysis of HL genes in hair cells, supporting cells, and Schwann cells of humans and mice. The blue represents the genes with HL phenotypes in mice, while orange indicates genes without HL phenotypes in mice. The y-axis represents the relative expression differences between humans and mice in different cell types. (B) The correlation of gene expression between humans and mice, categorized by cell type and HL phenotype. Genes with no expression in human or mouse were excluded. The blue represents the genes with HL phenotypes in mice, while orange indicates genes without HL phenotypes in mice.


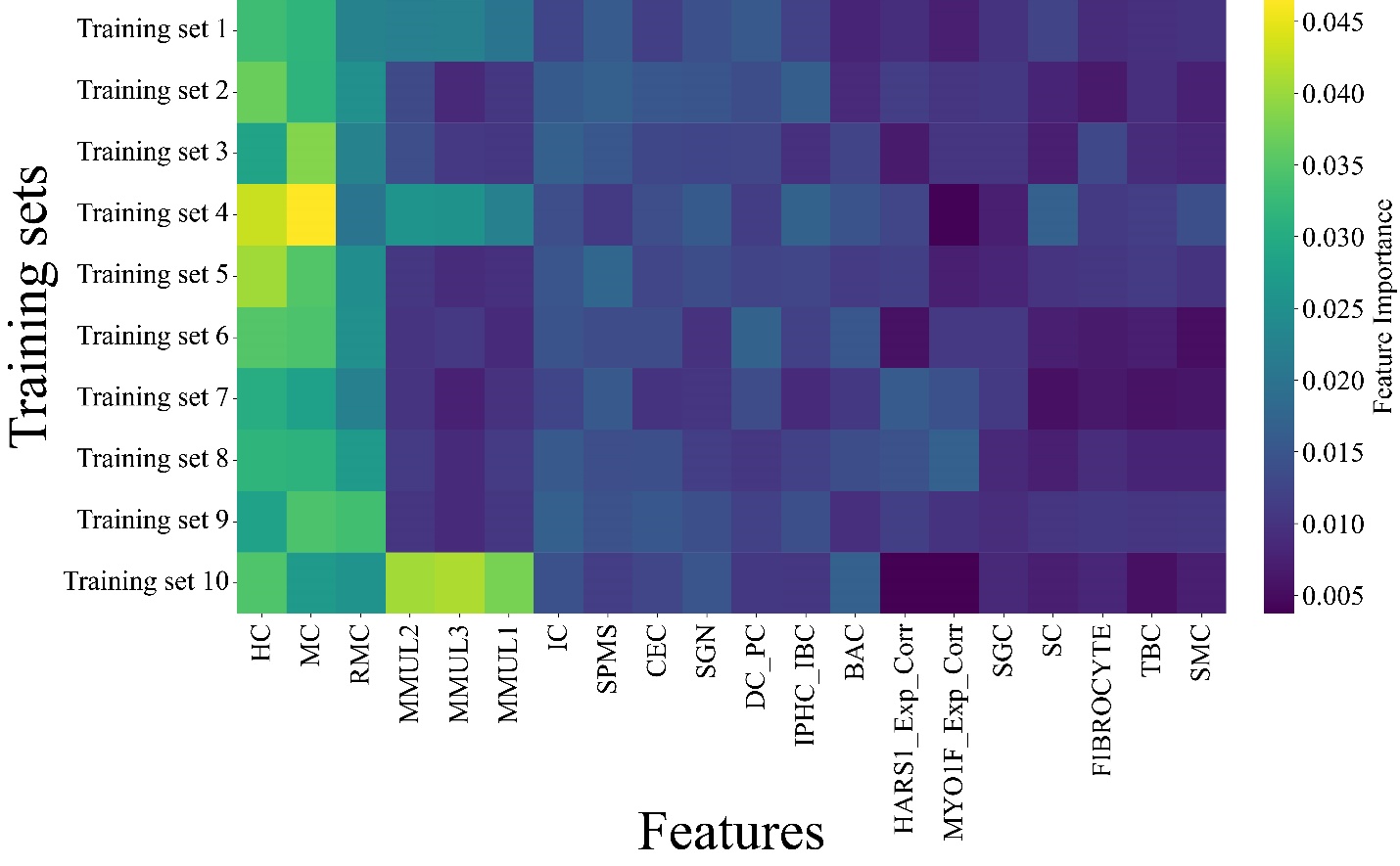


**Supplemental Figure S10:** Weights of top 20 feature across 10 training sets.

The color gradient from purple to yellow represents increasing feature importance as calculated by the Random Forest classifiers, with yellow indicating higher importance. Features are ordered in descending order based on their mean importances across all training sets. List of Abbreviations: HC, Hair cell; MC, Marginal cell; RMC, cells in Reissner’s membrane; MMUL1-3, rhesus macaque sample 1-3; IC, Intermediate cell; SPMS, sensory perception of mechanical stimulus; CEC, Capillary endothelial cell; SGN, Spiral ganglion neuron; DC_PC, Deiter cell and pillar cell; IPhC_IBC, Inner phalangeal cell/inner border cell; BaC, Basal cell; *HARS1*_Exp_Corr: Correlation coefficient of *HARS1* gene expression with other genes; *MYO1F*_Exp_Corr: Correlation coefficient of *MYO1F* gene expression with other genes; SGC, Satellite glial cell; SC, Schwann cell; TBC, Tympanic border cell; SMC, Smooth muscle cell

Supplementary Tables:

Supplementary Table S1: The annotation information of HL genes

Supplementary Table S2: Public data sources in GDC

Supplementary Table S3: Mutational landscape of HL genes

Supplementary Table S4: Analysis of mutational hotspots enriched with pathogenic missense variant

Supplementary Table S5: The mouse phenotype annotations and references for HL genes

Supplementary Table S6: Selected Features for ML model identifying candidate HL genes

Supplementary Table S7: Identification of 221 candidate HL gene using the ML model
